# Dimer asymmetry in signaling of blue light sensor histidine kinases

**DOI:** 10.1126/sciadv.aed8943

**Published:** 2026-07-01

**Authors:** Vladimir Arinkin, Andreas M. Stadler, Stefanie S.M. Meier, Karl-Erich Jaeger, Andreas Möglich, Ulrich Krauss, Renu Batra-Safferling

**Affiliations:** ^1^ Institut für Biologische Informationsprozesse (IBI), Strukturbiochemie (IBI-7), Forschungszentrum Jülich, 52425 Jülich, Germany.; ^2^ Neutron Scattering and Soft Matter (JCNS-1), Forschungszentrum Jülich GmbH, 52425 Jülich, Germany.; ^3^ Institute of Physical Chemistry, RWTH Aachen University, Landoltweg 2, 52056 Aachen, Germany.; ^4^ Department of Biochemistry, University of Bayreuth, 95447 Bayreuth, Germany.; ^5^ Institut für Molekulare Enzymtechnologie, Heinrich-Heine-Universität Düsseldorf, Forschungszentrum Jülich GmbH, 52425 Jülich, Germany.; ^6^ Institut für Bio- und Geowissenschaften (IBG), Biotechnologie (IBG-1), Forschungszentrum Jülich GmbH, 52425 Jülich, Germany.

## Abstract

Photoreceptor sensory histidine kinases (SHKs) couple light absorption to conformational changes regulating two-component signaling. Despite their importance and widespread use in optogenetics, the underlying structural signaling mechanisms remain poorly understood. Here, we engineered dimeric SHKs based on *Pseudomonas putida* short light-oxygen-voltage (LOV) proteins, determined their crystal structures, and investigated their signaling mechanisms. Regardless of illumination, the structures adopted a light-state like LOV-LOV dimer with symmetric/straight kinase modules. In contrast, small-angle x-ray scattering together with functional assays revealed pronounced light-dependent rearrangements in solution and allowed the assignment of the kinase-ON dark state to an asymmetric/kinked conformation, whereas the light state adopts a symmetric/straight structure. Comparative analyses of natural and engineered SHKs identified conserved motifs linking light-induced LOV domain rotation to kinase activity. The findings highlight the central role of dimer asymmetry and flexibility in SHK signaling, thereby not least informing the engineering of new light-responsive signaling systems.

## Teaser

Light rearranges the histidine kinase dimer, from an asymmetric kinase-competent to a symmetric phosphatase-competent state.

## INTRODUCTION

Protein phosphorylation is a ubiquitous regulatory process in living organisms, central to numerous cell signaling pathways. Mediated by kinases, it involves transferring a phosphoryl group from adenosine triphosphate (ATP) to amino acids such as serine/threonine (serine/threonine kinases), tyrosine (tyrosine kinases), or histidine [(histidine kinases (HKs)]. In prokaryotes, sensory HKs (SHKs), together with response regulators (RRs), constitute so-called two-component systems (TCSs). Frequently, the RR works as a transcription factor that controls gene expression in a stimulus-dependent manner. While TCSs are widespread in bacteria and archaea, they have also been identified in a few eukaryotes such as plants and yeasts (*1*, *2*). Bacterial TCSs are known to regulate a myriad of cellular processes, including nutrient acquisition; adaptation to environmental changes of, e.g., temperature, light, osmotic pressure, and pH; as well as stress responses, cell motility, development, pathogenicity, drug resistance/tolerance, and intercellular communication (*3*–*11*). One generally distinguishes canonical membrane-bound SHKs (*12*–*17*) and soluble, cytoplasmic SHKs (*8*–*11*, *18*–*21*). SHKs are typically homodimeric and feature an N-terminal sensor module tailored to specific stimuli and a C-terminal HK effector module, which comprises dimerization/histidine phosphotransfer (DHp) and catalytic/ATP-binding (CA) domains. The stimulus is detected by the sensor domain (or module) initiating autophosphorylation of the HK module, which is associated with the transfer of a phosphoryl group from ATP, bound in the CA domain, to a conserved histidine in the DHp domain. The CA domain harbors several highly conserved sequence motifs (known as the N, F, G1, and G2 regions) that together form the ATP binding and catalytic core of HKs crucial for autophosphorylation and the conformational transitions required for phosphoryl transfer (*6*) to an aspartate residue of a cognate RR, which for many TCSs controls gene expression in phosphorylation-dependent fashion. SHKs also regulate “active” RR levels via dephosphorylation (phosphatase activity) of the RR, forming a cycle that fine-tunes cellular responses. Depending on signal, one of the two activities outweighs the other, and the SHK thus serves as either a net kinase or net phosphatase on its cognate RR (*22*, *23*).

**Fig. 1.**
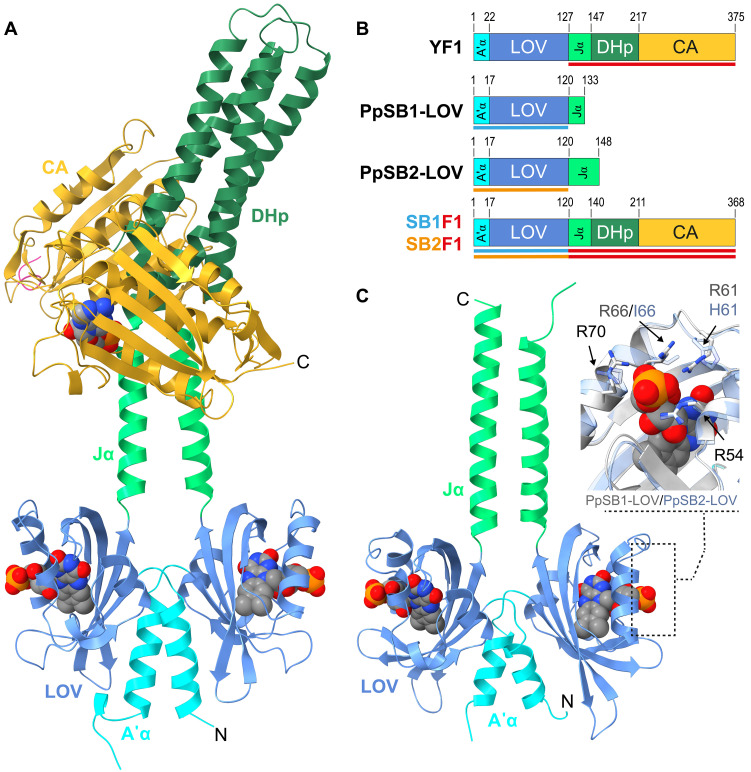
Domain architectures of the designed SHKs and structural similarity between YF1 and PpSB2-LOV. Dark-state structure (**A**) and multidomain architecture (**B**) of YF1 with the A′α (residues 1 to 22) in cyan, LOV domain (residues 23 to 127) in blue, Jα linker (residues 127 to 147) in light green, the rest of the DHp domain (residues 148 to 217) in dark green and CA domain (residues 218 to 375) in gold. N and C termini are highlighted in one of the two chains, respectively. The LOV domain FMN chromophore and the one adenosine diphosphate (ADP) molecule bound in one YF1 CA domain are shown as VdW spheres with carbon in gray, nitrogen in blue, oxygen in red, and phosphorous in orange. SB1F1 and SB2F1 were designed by exchanging the YtvA LOV domain of YF1 (residues 1 to 127) by the LOV domains of PpSB1-LOV and PpSB2-LOV (residues 1 to 120), respectively. The colored lines below the domains indicate the origin of respective domains. The dark-state structure of PpSB2-LOV [PDB-ID: 7A6P, (*53*)] is shown in (**C**). Compared to PpSB2-LOV, PpSB1-LOV has a slightly shorter C-terminal Jα helix. A multiple sequence alignment of YF1, SB1F1, and SB2F1 is shown in fig. S1. The inset depicts the region around the FMN phosphate moiety in PpSB1-LOV (gray) and PpSB2-LOV (transparent blue). The arginine cluster (R54, R61, R66, and R70) interacting with the FMN phosphate in PpSB1-LOV and the corresponding residues of PpSB2-LOV are shown in stick representation with C in gray, O in red, and N in blue for PpSB1-LOV in light blue and blue for the PpSB2-LOV. The two arginine residues R66 and R61 present only in PpSB1-LOV are encircled by a red dotted line.

A subset of SHKs are photoreceptors (*24*) that respond to different wavelengths of the visible to near-infrared spectrum, with red- to near-infrared light sensing bacteriophytochromes (*25*–*30*) and blue light sensing light-oxygen-voltage (LOV) photoreceptors (*8*–*11*) as the most widely studied systems. LOV-HKs represent interesting model systems for the study of SHK signaling due to several reasons. First, the biological function of several LOV-HKs has been elucidated, with the systems playing an important role in bacterial physiology, ranging from regulating bacterial virulence and pathogenicity to cell-cell/cell-surface attachment and control of sulfur metabolism (*8*–*10*, *31*, *32*). Second, HK function can be triggered by short pulses of light with high spatiotemporal resolution, enabling, in principle, the straightforward study of HK structure and function (*33*–*35*). Last, photoreceptor HKs including LOV-HKs and phytochrome-based systems have been widely used in optogenetics to regulate biological processes with light, such as gene expression in bacteria and yeast (*36*–*40*). In these studies, either naturally existing photoreceptor TCSs are used (*40*) or the photoreceptor sensory module [e.g., the LOV sensory domain or the phytochrome photosensory core module (PCM) consisting of PAS-GAF-PHY domains] is fused to a well-characterized HK effector module, consisting of DHp and CA domains (*23*, *37*, *38*). The first described blue light–dependent engineered SHK is the chimeric protein YF1 (*23*) comprising the N-terminal sensory domain of the *Bacillus subtilis* YtvA photoreceptor (*41*) and the DHp and CA domains of the FixL HK of *Bradyrhizobium japonicum* (*42*). YF1 phosphorylates its cognate RR FixJ in the dark, whereas upon blue light illumination, the phosphatase activity of YF1 is enhanced, thereby reducing net kinase activity by more than 1000-fold (*23*). When the YF1/FixJ TCS is used to control gene expression in *Escherichia coli* in a light-dependent manner, this translates into high gene expression in the dark and low expression in blue light (*23*, *39*). YF1 function was shown to depend on the LOV photocycle (*23*) that involves the transient formation of a FMN-cysteinyl thioadduct between a conserved cysteine in the LOV domain (residue C62 in YF1 that is part of the conserved GXNCRFLQ sequence motif characteristic for LOV domains) and the flavin mononucleotide (FMN)-C4a atom (*43*, *44*). The formation of the light or adduct state is commonly associated with structural changes in the LOV domain that are transmitted to fused effector domains such as, e.g., the HK DHp/CA domains of YF1. Signal relay between the sensor LOV and effector domains hereby often involves structural rearrangements of the LOV domain N- and C-terminal flanking helices (A′α and Jα; Fig. 1) (*45*–*48*). In YF1, the Jα helix is conjoined with the α1 helix of the DHp domain (fig. S1). Structural changes in the YF1 LOV-LOV dimer are likely coupled to conformational transitions of the C-terminal HK module via the conserved D125-I126-T127 (DIT) motif (found at the LOV-Jα junction) (*33*). Thus, consistent with previous naming conventions (*33*) and for the sake of clarity, we here refer to the helix coupled to and extending from the LOV core in YF1 and related constructs as the Jα helix. In the dark, the LOV photoreceptor thermally returns to its dark state (dark recovery), which, depending on the protein, can take between seconds and hours (*45*, *49*).

Despite fulfilling important functions in bacteria and their widespread use as optogenetic tools, high-resolution structures of full-length SHKs remain scarce, limiting our understanding of the signal-dependent structural response underlying HK function and phosphorelay. In this context, soluble photoreceptor SHKs provide tractable paradigms for dissecting the structural principles of SHK signaling. At present, three structures of LOV SHKs have been reported (*33*–*35*), yet no full-length structure of a dimeric LOV-HK has been determined in both its dark and light states, and the correlation of these states with kinase activity (i.e., ON or OFF states) remains elusive. Reasons for the lack of crystallization success likely include the flexibility of the HK (DHp-CA) module in either of the two states (*34*) and/or too fast dark recovery of the LOV-HK, which in many cases precludes the crystallization of the protein in the light state (*33*, *45*, *50*, *51*). To this end, we have previously shown that the exceedingly slow dark recovery (τ_rec_ ~2400 min at 20°C) (*52*) of the LOV protein PpSB1-LOV of *Pseudomonas putida* allows crystallization in both the dark and light states (*45*, *47*), while its fast-recovering counterpart PpSB2-LOV (τ_rec_ ~ 2 min at 20°C) (*52*) could only be crystallized in the dark state (*53*). Both PpSB1-LOV and PpSB2-LOV are part of the so-called short LOV protein family that do not contain a fused effector domain but instead contain short N- and C-terminal helical extensions (*45*, *52*). Given the structural similarities between the LOV domain dimer in YF1 and the PpSB1-LOV/PpSB2-LOV dimer (Fig. 1), we reasoned that the replacement of the YtvA LOV domain in YF1 by the corresponding LOV domain of PpSB1-/PpSB2-LOV enables the construction of functional light-dependent HKs, with very slow and fast dark recovery kinetics, respectively. Not only can this approach yield optogenetic tools for practical applications, but it also increases the likelihood of crystallizing both the dark and light states.

In this study, we present the engineering and functional characterization of YF1-like sensor HKs derived from *P. putida* PpSB1/PpSB2-LOV that display very different recovery kinetics lasting days for the SB1F1 protein and only minutes for SB2F1 and its slower reverting variant SB2F1-I66R. Functional assays confirm that both SB1F1 and SB2F1 operate as light-repressed HKs as YF1 does. We successfully determined the dark-state structure of SB2F1 and both the light- and dark-state structures of the slower cycling SB2F1-I66R variant. Our findings, complemented by solution x-ray scattering studies, suggest that the activation of homodimeric LOV-SHKs is driven by light-induced transitions between symmetric and asymmetric structures, resulting in net kinase (kinase ON) or net phosphatase (kinase OFF) activity, respectively. This structural switching suggests a mechanistic basis for how LOV-SHKs integrate light signals into opposing enzymatic outputs.

## RESULTS

### Construction of sensor HKs based on the PpSB1-/PpSB2-LOV modules

To construct SB1F1 and SB2F1, we replaced residues 1 to 127 of YF1 (comprising the A′α helix, the LOV core domain up to the YF1 DIT sequence motif (D125-I126-T127) with the corresponding parts of PpSB1-LOV (residues 1 to 120) and PpSB2-LOV (residues 1 to 120) (Fig. 1 and fig. S1). The I66R mutation, which slows recovery of the parental PpSB2-LOV protein by about sevenfold (*52*) (marked with an asterisk in fig. S1) was introduced in SB2F1 to decelerate its recovery, with the reasoning that this could facilitate the crystallization of the protein in both the dark and light states. In the slow-reverting homolog PpSB1-LOV, R66 is part of a four-arginine cluster (R54, R61, R66, and R70) that forms a salt-bridge network around the FMN phosphate, previously implicated in slowing LOV dark recovery (*45*, *52*, *54*). In contrast, PpSB2-LOV contains only two phosphate-interacting arginine residues (R54 and R70), a hydrophobic isoleucine at position 66 and a histidine at position 61 (Fig. 1C, inset). Thus, the I66R mutation is expected to extend the arginine cluster interacting with FMN, thereby potentially stabilizing the LOV domain light state by constraining the FMN orientation in the light-state geometry and increasing the energetic barrier for adduct decay.

### SB1F1 and SB2F1 are functional light-dependent HKs with disparate dark recovery kinetics

**Fig. 2.**
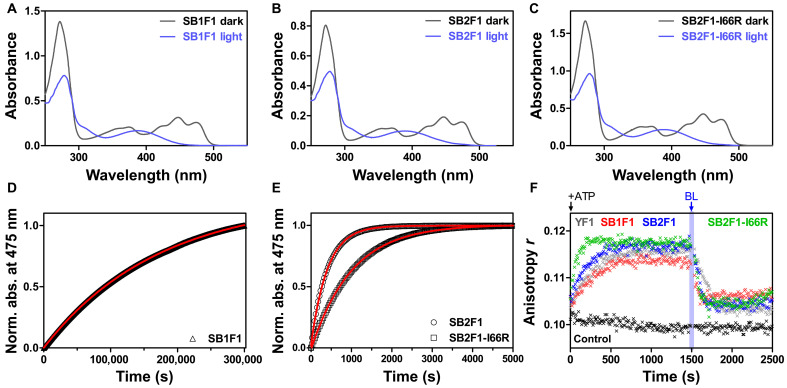
Functional characterization of SB1F1, SB2F1, and SB2F1-I66R. (**A** to **C**) Dark-state (dark gray line) and light-state (light blue line) absorbance spectra of SB1F1 (A), SB2F1 (B), and SB2F1-I66R (C). Light-state spectra were recorded after illuminating the purified protein for 30 s with a blue LED. (**D**) Dark recovery time trace recorded for the SB1F1 protein after 30-s blue light illumination. For SB1F1, recovery remained incomplete after 5000 min, but measurements had to be stopped due to experimental constraints. (**E**) Dark recovery time trace recorded for the SB2F1 and SB2F1-I66R protein after 30-s blue light illumination. Data shown in (D) and (E) are normalized to aid visualization. All dark recovery measurements were performed at 20°C. (**F**) Histidine-kinase activity assays. Dark equilibrated samples of SB1F1 (red), SB2F1 (blue), SB2F1-I66R (green), and YF1 (gray) were incubated in the dark with FixJ and a double-stranded DNA molecule containing the phospho-FixJ operator site and labeled at the 5′ end with a TAMRA fluorophore (fig. S2A). To start the kinase reaction, ATP was added and TAMRA fluorescence anisotropy was monitored over time. An increase in anisotropy reflects kinase activity, i.e., resulting in the formation of phospho-FixJ and its binding to the DNA. Subsequent illumination with blue light (indicated by a blue bar) caused a decrease of the anisotropy, consistent with net phosphatase activity. A control reaction (black crosses) was performed that contained only the TAMRA-labeled DNA substrate but no SHK/RR. All experiments were performed in triplicate with consistent results (see fig. S2, B to E).

SB1F1, SB2F1, and SB2F1-I66R were heterologously produced in *E. coli* BL21(DE3) and purified to homogeneity. Ultraviolet/visible (UV-Vis) spectra of the purified proteins (Fig. 2, A to C) in their dark state showed typical LOV-protein–like spectra with the main absorbance bands in the visible regions centered at 447 nm and ~360 nm (*43*, *44*, *49*).

In all cases, illumination with blue light resulted in the loss of the absorbance at 447 nm and the formation of a new maximum at about 390 nm, indicative of canonical LOV photochemistry involving FMN-cysteinyl adduct formation (*43*, *44*, *49*). The recovery of the dark state of the three proteins was followed by recording the absorbance at 475 nm after illumination (Fig. 2, D and E). As expected, from the dark recovery kinetics of the corresponding parent LOV-proteins (*52*), SB1F1 and SB2F1 showed very slow (SB1F1; τ_rec_ ~ 2850 min) and fast (SB2F1; τ_rec_ = 7.0 ± 0.5 min) dark recovery kinetics, respectively. Similar to earlier studies, the I66R mutation slowed down the recovery of SB2F1 (*52*) by a factor of 2.5 (SB2F1-I66R; τ_rec_ = 17.3 ± 0.8 min).

To probe the functionality of the designed LOV-HKs, we made use of a fluorescence anisotropy assay that continuously monitors the catalytic activity and the light response of LOV-HKs such as YF1 (fig. S2A) (*55*). In the dark and in the presence of ATP, YF1 phosphorylates FixJ, triggering its dimerization and binding to a 5′-tetramethylrhodamine (TAMRA)–labeled double-stranded DNA oligonucleotide that contains the FixK2 operator sequence. Binding slows DNA rotational diffusion and increases the fluorescence anisotropy of the TAMRA label. Blue light illumination results in the formation of the metastable YF1 light state that persists after the light is switched off and exhibits enhanced phosphatase activity, resulting in FixJ dephosphorylation, DNA dissociation, and reduced fluorescence anisotropy. We initially used YF1 as a reference. As expected, upon the addition of ATP, dark-adapted YF1 showed increased fluorescence anisotropy (Fig. 2F and fig. S2B; gray symbols), whereas illumination with blue light triggered a rapid reduction of the fluorescence anisotropy, reflecting net phosphatase activity of YF1 in its light state, as previously reported (*23*, *55*). All LOV-HKs designed here showed very similar behavior, i.e., net kinase activity in the dark and net phosphatase activity upon blue light illumination, albeit with slightly different kinetics and amplitudes (Fig. 2F and fig. S2, C to E, red, green, and blue symbols, respectively). This, together with the spectroscopic data, reporting on LOV-photocycle integrity, demonstrates that SB1F1, SB2F1, and SB2F1-I66R are functional LOV-HKs, with very similar catalytic properties as YF1, although with altered dark recovery kinetics.

### Crystallization and dark-state structure of SB2F1

**Fig. 3.**
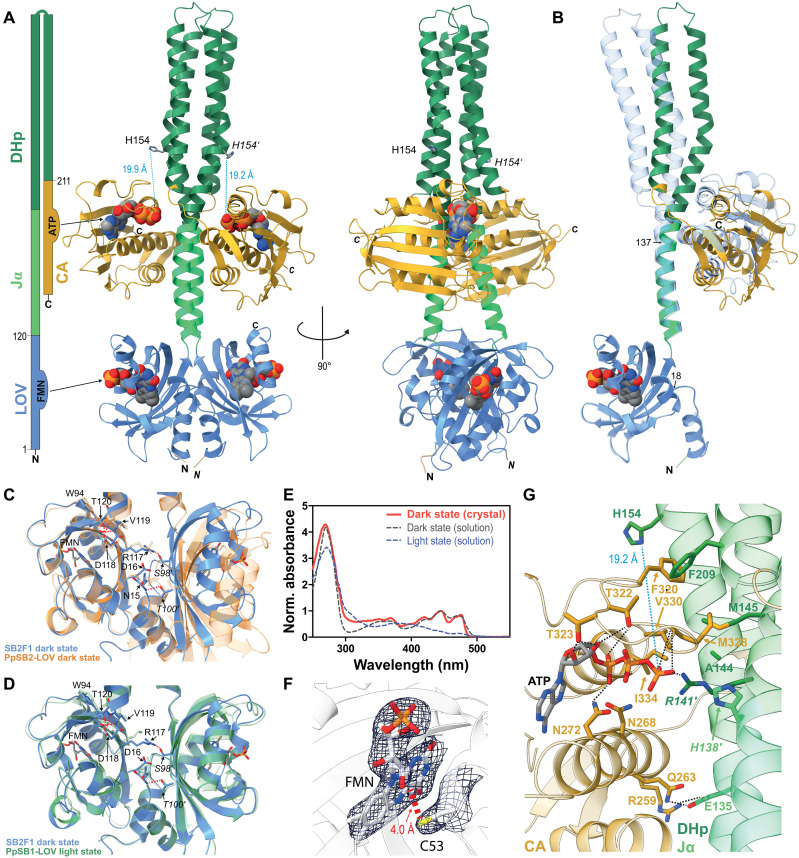
SB2F1 dark-state structure. (**A**) Crystal structure of SB2F1 bound to ATP, shown as a ribbon diagram with LOV (blue), DHp including Jα-helix portion (light and dark green), and CA (gold) domains. FMN and ATP are in spheres representation; H154 (phospho-acceptor) is shown as sticks. Atom colors: C in gray, O in red, N in blue, and P in orange. (**B**) Superposition of SB2F1 chain A [colored as in (A) with chain B (transparent light blue)], aligned over residues 17 to 138 to highlight divergence beyond the DHp domain. (**C** and **D**) Superpositions of the SB2F1 dark-state LOV dimer (blue) with the dark-state PpSB2-LOV (orange, C) and light-state PpSB1-LOV (green, D) dimers. FMN is shown as sticks; dimers were aligned over residues 1 to 120. Key inter- and intrachain interactions are shown in stick representation with C in light blue, O in red, N in blue, and P in orange; for SB2F1, light orange and light green for the PpSB2-LOV dark state (C) and PpSB1-LOV light state (D), respectively. (**E**) Single-crystal microspectrometry data of dark-grown SB2F1 crystals (red) compared to SB2F1 in solution (dark state: dashed gray; light state: dashed blue), normalized at 450 nm. (**F**) σ-A weighted 2mFo-DFc electron density map of the FMN and residue C53 of SB2F1 contoured at 1.0 σ. (**G**) SB2F1 dark-state structure as in (A), highlighting CA and DHp residues interacting with ATP and mediating interdomain contacts. H bonds (<3.2 Å) shown as black dashed lines; H154^…^ATP-γ-P distance shown as cyan dashed line in (A) and (G). Interacting residues from the other chain are italicized throughout.

We reasoned that the very slow dark recovery of SB1F1 (τ_rec_ ~ 2850 min) could facilitate crystallization in both the light and dark states. This strategy had previously proven effective with the short PpSB1-LOV protein (τ_rec_ ~ 2400 min), for which we successfully obtained crystal structures in both states (*45*, *47*). Unfortunately, we were unable to obtain diffraction quality crystals for SB1F1. We therefore next focused on the fast-reverting SB2F1 protein (τ_rec_ = 7.0 ± 0.5 min). Native crystals were obtained using the sitting-drop vapor diffusion method (for details, see Materials and Methods). However, despite the high sequence similarity between SB2F1 and the YF1 protein (Fig. 1 and fig. S1), phasing attempts by molecular replacement using the YF1 model as a template were unsuccessful, already hinting at substantial structural differences. We hence obtained SeMet-substituted crystals for single-wavelength dispersion (SAD) phasing. As detailed in Materials and Methods, SB2F1 crystals obtained in the presence of ATP and MgCl_2_ were grown in the dark in space group P3_1_21 and diffracted to a resolution of 2.45 Å (table S1). A smaller subset of datasets (<5%) exhibited crystals in the closely related P3_2_21 space group, where both crystal forms originated from the same crystallization drop [table S1; Protein Data Bank (PDB) ID: 8A3U]. The structural superposition of the two models demonstrated that they are nearly identical, with a Cα root mean square deviation (RMSD) of 1.09 Å over residues 1 to 365 (table S2). Given that all other structures analyzed in this study adopt the P3_1_21 space group, the SB2F1 dark-state structure corresponding to PDB ID: 8A6X is used for subsequent discussion and comparison. The SB2F1 dark-state structure contains two protein molecules per asymmetric unit that form a long, rod-shaped homodimer (Fig. 3A).

Residues 1 to 365 of 368 were resolved in both monomers. In both protein chains, the LOV domain contains a noncovalently bound FMN molecule, while the CA domains bind ATP. Because of the relatively poor resolution in several residues of the CA domain, its refinement was restrained using the YF1 crystal structure, which has the same sequence. The superposition of the SB2F1 constituent monomers reveals moderate structural differences, reflecting a slightly asymmetry within the altogether largely C-symmetric dimer. The alignment of residues 17 to 138 (LOV core and DHp domain up to H138) highlights some degree of tertiary structure differences caused by DHp helix bending beyond H138 (Fig. 3B).

The superposition of the dark-state dimer of the short LOV protein PpSB2-LOV on the SB2F1 dark-state LOV-LOV dimer results in a poor alignment (Cα RMSD over residues 1 to 118; 3.23 Å) (Fig. 3C). In contrast, superposition with the light-state structure of PpSB1-LOV yields a substantially better fit (Cα RMSD over residues 1 to 118: 1.05 Å) (Fig. 3D and table S2). Hence, in terms of the overall LOV-LOV dimer, the SB2F1 dark-state structure seems to be much closer to a light-state structure [here represented by the light-state structure of PpSB1-LOV, PDB-ID: 3SW1 (*45*); since no PpSB2-LOV light-state structure is available] than to the corresponding PpSB2-LOV dark-state dimer [PDB-ID: 7A6P, (*53*)]. This apparent conundrum is also reflected in intra- and interchain interactions centered around the LOV-LOV dimer interface, which is relevant for signaling. In SB2F1 those interactions include interchain contacts between N15^…^*T100*′, R117^…^*S98*′ (interacting residues from the symmetric dimer chain are italicized and marked with prime notation) and intrachain contacts between residues of the DVT (D118-V119-T120) motif and W94 (D118^…^T120, V119^…^W94) (compare Fig. 3, C and D). A similar interaction pattern is present in the PpSB1-LOV light-state structure and is seen in molecular dynamics simulations (*56*, *57*). In contrast, the pattern is changed in the corresponding dark-state structure of PpSB1-LOV that displays an N15^…^*S98′* interaction and an R117^…^D16 interaction, while the interactions among the residues of the DVT motif remain undisturbed (*47*). To verify that the crystals grown under dark conditions remained in the dark state during preparation, single-crystal microspectrometry was performed on the dark-grown SB2F1 crystals immediately before x-ray diffraction data collection. The results show a canonical dark-state spectrum for the crystal, consistent with the spectrum observed in solution (Fig. 3E). In addition, in the corresponding SB2F1 dark-state 2mFo-DFc electron density map, no continuous density is observed between the FMN-C4a atom and the C53-SG atom, with C53 adopting a single conformation pointing away from the FMN-C4a atom (FMN-C4a^…^SG-C53; 4.0 Å) that is commonly associated with the structures of dark-adapted LOV receptors (Fig. 3F) (*47*, *58*–*60*). Thus, both single-crystal microspectrometry and the electron density around the FMN/C53 region indicate that the FMN molecules in the individual LOV domains are in a dark-adapted state, with no covalent bond between the FMN-C4a atom and C53, although the LOV-LOV dimer arrangement within SB2F1 more closely resembles the conformation seen for the parental PpSB1-LOV light-state dimer (Fig. 3D).

In both CA domains of the SB2F1 dimer (Fig. 3, A and G), ATP can be fitted into the electron density map (fig. S3). The distance between the phospho-accepting H154 in the DHp domain and the ATP γ-phosphate is 19.2 Å in chain A and 19.9 Å in chain B (Fig. 3A), and thus too far to enable phosphotransfer (in cis) between ATP in the CA domain and H154. Therefore, the HK domain structure likely corresponds to a “kinase OFF” state. The main interactions within the CA and DHp domains involve the ATP molecule, highlighting key contacts that stabilize the CA-DHp interface (Fig. 3G). The ATP molecule is coordinated by residues within the conserved N, F, G1, and G2 sequence regions (*61*), involving H-bonding interactions to the side chains of N272, T322, T323, and the M328 and G329 backbone. In the ATP binding pocket of the SB2F1 CA domain, there is continuous electron density between N268, N272, and the phosphate groups of ATP, even at high σ contouring of the 2mFo-DFc electron density maps (fig. S3A). The mFo-DFc map, contoured at 3σ, shows some additional density not covered by the model (fig. S3A). Superposition with the structurally similar kinase domain, WalK (fig. S3B) (*62*), showed that WalK with bound ATP has a magnesium ion located approximately in this continuous density. Although the Mg^2+^ ion was not modeled in the SB2F1 structure due to the low resolution of the CA domain maps, the strong alignment of ATP and the kinase domains between the two structures suggests that an Mg^2+^ ion likely contributes to ATP binding and catalysis in SB2F1 as well. The interaction between the CA and DHp domains (Fig. 3G) is mostly mediated by hydrophobic interactions (F320^…^F209 and M328^…^M145 and centered around I334, V330, and A144), hydrogen bonds (E135^…^Q263), and salt bridges (E135^…^R259). Residue R141 on the DHp domain interacts with the ATP γ-phosphate.

### Illumination of dark-grown SB2F1 crystals only results in local photoactivation

The crystallization of SB2F1 under continuous blue light illumination yielded crystals, which, however, diffracted only to poor resolution (lower than 10 Å). Therefore, SB2F1 crystals grown in the dark in the presence of ATP and MgCl_2_ were illuminated with blue light before data collection to assess light-induced structural changes; this structure is referred to as SB2F1 “illuminated state.” Because of crystal lattice constraints, it does not reflect the “true” light state, as previously observed for other LOV proteins (*47*). When we compare the structures of dark-grown SB2F1 and “illuminated” SB2F1, only minor global differences can be observed (fig. S4) (Cα RMSD of 0.64 Å superimposed over LOV-LOV dimer). When the two structures are superimposed via chain A of the LOV-LOV dimer, the differences become visible (fig. S4A). These include a slight rotation of the chain B LOV dimer relative to chain A accompanied by a minor displacement of the DHp domains, which is amplified to both CA domains (fig. S4A), corroborated by increased residue-wise RMSDs for the CA domains (fig. S4B). While single-crystal microspectrometry data verifies light-state like UV-Vis spectra for the illuminated crystal (fig. S4C), the corresponding 2mFo-DFc electron density map did not show any continuous electron density between FMN-C4a and C53-SG (fig. S4D), which might be attributed to x-ray–induced radiation damage, as described for other LOV protein structures (*45*, *63*–*65*). Local photoactivation in the crystal is also indicated by the rotation of C53 to fully occupy the position atop the flavin C4a atom in one of the chains, accompanied by a flipping of Q116, which are hallmarks of LOV photoactivation (fig. S4D) (*55*, *66*, *67*).

### Dark- and light-state SHK structures can be obtained for the slower cycling SB2F1-I66R variant

**Fig. 4.**
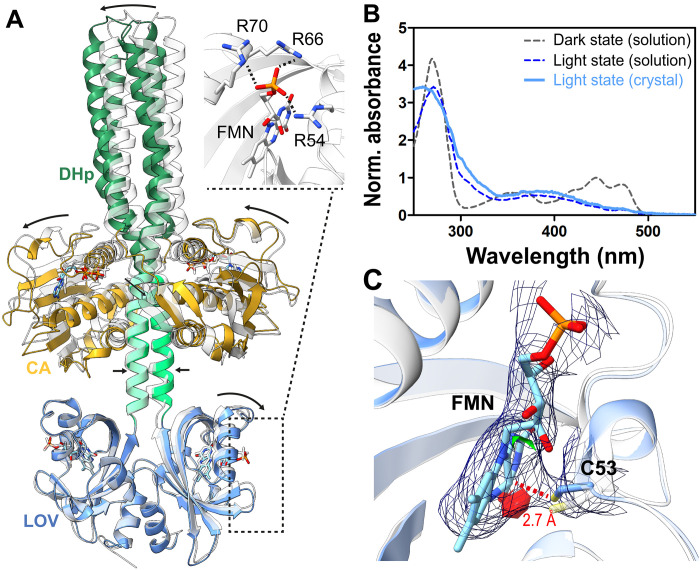
Comparison of SB2F1-I66R dark-state and SB2F1-I66R light-state structure. (**A**) Crystal structures of the SB2F1-I66R dark-state structure (transparent gray ribbon) superimposed with the SB2F1-I66R light-state structure (solid ribbon) with the domains colored as described in Fig. 3. The inset depicts the region surrounding the FMN-phosphate of the SB2F1-I66R dark state, highlighting the interaction between the FMN phosphate moiety, the conserved arginine’s (R54, R77) and the introduced R66 (FMN and side chains in stick representation; dashed lines depict interatomic distances <3.2 Å). FMN and ATP in stick representation with C in gray, O in red, N in blue, and P in orange in the light-state structure and are kept gray transparent in for the dark-state structures. Structures superimposed via chain A of the LOV-LOV dimer. Arrows mark the direction of dark-to-light state movements. (**B**) Single-crystal microspectrometry data of SB2F1-I66R light-state crystal (solid light blue line) in comparison to SB2F1 solution dark- (dashed gray line) and light-state spectra (dashed blue line). All spectra were normalized to the 450-nm (dark state) and 390-nm (light state) absorbance band, respectively. (**C**) 2mFo-DFc electron density map of the FMN chromophore and C53 of the SB2F1-I66R light-state structure contoured at 1.0 σ. The corresponding mFo-DFc map is shown as green (3.0 σ) and red (−3.0 σ) colored surface 2 Å from the FMN and C53.

Next, to enable crystallization of SB2F1 under continuous blue light illumination, the SB2F1-I66R variant was used, which has a prolonged adduct-state lifetime of τ_rec_ = 17.3 ± 0.8 min (Fig. 2E) as compared to SB2F1 (SB2F1; τ_rec_ = 7.0 ± 0.5 min), potentially enhancing protein crystallization in the light state while maintaining SHK functionality (Fig. 2F). SB2F1-I66R with bound ATP could be crystallized in the dark and under continuous blue light illumination (light state) (table S1). For both conditions, crystals were obtained in space group P3_1_21 diffracting up to 2.71 and 3.145 Å, respectively. Strikingly, both crystals exhibited closely similar unit-cell dimensions despite being obtained in very different crystallization conditions. As expected from structural studies of the homologous LOV domain of the slow-reverting PpSB1-LOV protein, the newly introduced R66 forms an additional interaction with the FMN phosphate, thereby extending the R54/R70 arginine cluster (Fig. 4A, inset). Since we observed a light-state like LOV-LOV dimer arrangement in the SB2F1 dark-state structure (see above), we first assessed whether the SB2F1-I66R dark-state structure also adopts a light-state like LOV-LOV dimer. As observed before for SB2F1, the superposition of the SB2F1-I66R dark-state structure with the PpSB2-LOV dark-state dimer yields a much worse fit (Cα RMSD over residues 1 to 118; 3.23 Å) than the superposition with the PpSB1-LOV light-state dimer (Cα RMSD over residues 1 to 119; 1.05 Å) (fig. S5 and table S2). The superposition of the SB2F1-I66R light-state structure with the PpSB1-LOV light-state dimer yields a low Cα RMSD of 0.86 Å (over residues 1 to 118), while the superposition with the PpSB2-LOV dark-state dimer yields a Cα RMSD of 3.09 Å (over residues 1 to 118), indicating that the overall arrangement of the LOV-LOV dimer does not change markedly in the SB2F1-I66R light-state structure as compared to the dark state. This can also be seen when the SB2F1-I66R dark- and light-state structures are superposed via chain A of the dark-state dimer (Fig. 4A). Likewise, in terms of the global dimer structure, the changes are modest. The most pronounced structural change is a slight displacement of the DHp domain away from the symmetry axis that renders the overall dimer slightly less symmetric in the light state (fig. S6).

To verify that the structure obtained from light-grown crystals represents a light-state conformation (inferred from FMN-Cys adduct formation), we recorded single-crystal microspectrometry data for the light-grown SB2F1-I66R crystal. The results confirm adduct formation in the crystal (Fig. 4B). We therefore modeled the FMN-C4a atom as *sp*3 hybridized, as also reported in our previous PpSB1-LOV and SBW25-LOV light-state structures (*45*, *68*). However, although the corresponding 2mFo-DFc electron density map shows continuous electron density between FMN-C4a and C53-SG (Fig. 4C), and the C53 side chain has moved to a position directly on top of the FMN-C4a atom, the FMN-C4a ^…^ C53-SG distance is with 2.7 Å, too long for a covalent bond. These observations may be attributed to x-ray–induced radiation damage, as reported also for other LOV protein crystal structures (*45*, *63*–*65*).

### SAXS studies in solution reveal light-dependent structural changes

Small-angle x-ray scattering (SAXS) is well-suited for investigating the structure of proteins and macromolecules in solution and thus complements crystallography. Notably, SAXS enables the determination of the oligomeric state in solution through an experimental estimate of the molecular mass. Moreover, SAXS provides low-resolution structural information on the overall shape and domain organization. While all the crystal structures of SB2F1 in the dark, illuminated and light states reveal a largely symmetric dimer, these structures do not allow an unambiguous assignment of the functional state of both the LOV-LOV dimer and the HK module. This is particularly relevant, as crystal packing effects can influence domain orientations, potentially obscuring conformations that are relevant in solution. To address these issues, we recorded solution SAXS data for SB2F1 in the presence of ATP and MgCl_2_, both in darkness and after illumination with blue light (table S3).

**Fig. 5.**
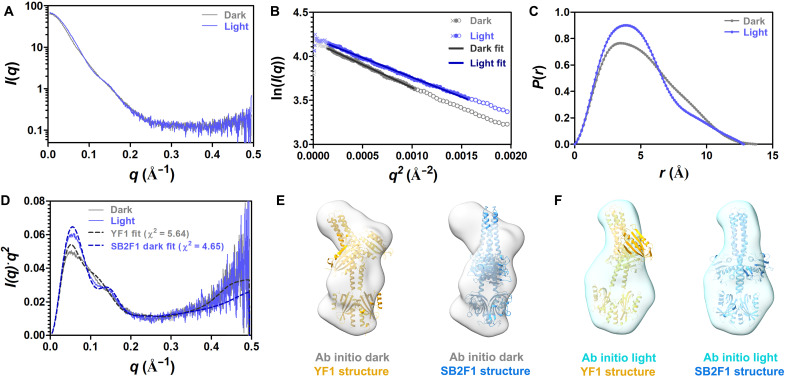
SAXS data obtained for ATP-bound SB2F1 in dark and light states. SAXS data of protein samples supplemented with 1.5 mM ATP and 2 mM MgCl_2_. (**A**) SAXS scattering curves and (**B**) Guinier plot for *q* × *R*_g_ < 1.3. Open symbols indicate data beyond the Guinier region. Data points in the low *q* region, represented by crosses, were excluded. Solid lines represent the Guinier fit. (**C**) Pair distribution function, *P*(*r*) and (**D**) Kratky plot and Crysol-based model evaluation. The theoretical scattering curves (gray and blue dashed lines) calculated from the YF1 dark-state structure [PDB-ID: 4GCZ; (*33*)] and SB2F1 dark-state structure [PDB ID: 7A6P, (*53*)] are shown. (**E** and **F**) Averaged and filtered ab initio models (transparent surface; gray: dark state, cyan: light state) superimposed with the YF1 dark-state structure [PDB-ID: 4GCZ; (*33*); orange cartoon] and SB2F1 dark-state structure (blue cartoon). For clarity, FMN and ADP/ATP ligands are not shown. Density map is shown at a contour level of 1σ and colored as indicated in the figure. Further details are shown in fig. S8.

The corresponding SAXS scattering data (Fig. 5, A and B) indicates that the samples are homogeneous and nonaggregating. SAXS and dynamic light scattering (DLS) measurements confirmed that SB2F1 (in the presence of ATP and MgCl_2_) is a dimer in both the dark and light state (table S3 and fig. S7). Analytical size exclusion chromatography (SEC), performed exclusively in the dark due to the rapid dark recovery of SB2F1, overestimates the molecular weight, likely because of the elongated, nonspherical shape of the molecule, but nevertheless confirms its dimeric quaternary structure. The Guinier analysis of the SAXS data (Fig. 5B) suggests that the dark state (*R*_g_ = 3.96 ± 0.01 nm) is slightly more extended compared to the light state (*R*_g_ = 3.73 ± 0.01 nm) (table S3). Large-scale conformational differences between the dark and light samples are visible from the pair distribution *P*(*r*) function and the Kratky plot (Fig. 5, C and D). To assess how the SB2F1 structure differs between crystal and solution, the theoretical scattering profiles calculated for the crystal structures were compared to experimental SAXS data.

Overall, neither of the theoretical scattering profiles of the crystal structures (irrespective of potential functional state or variant) fits the experimental scattering data recorded for ATP-bound SB2F1 in the dark state very well (χ*^2^* between 23.3 and 24.4; table S3). The theoretical scattering curve calculated for the YF1 dark-state structure provides a much better fit [χ^2^ = 5.64; Fig. 5D (gray dashed line) and table S3]. The opposite holds for the light-state SAXS data. Here, the theoretical scattering profile calculated for all SB2F1 structures provide a reasonably good fit (χ^2^ = 4.66 to 6.32; table S3) with the ATP-bound dark-state SB2F1 structure yielding the best fit [Fig. 5D (blue dashed line) and table S3]. In contrast, the YF1 structure exhibits a poorer fit (χ^2^ = 8.9; table S3).

Similar observations are made in the ab initio envelope models calculated from the corresponding SAXS scattering data (Fig. 5, E and F, and fig. S8). Strikingly, the envelope calculated for the dark-adapted protein exhibits a marked kink, whereas that determined for the light-adapted protein is largely symmetric. All SB2F1 structures (irrespective of assigned state) much better fit the light-state envelope (exemplarily shown for the SB2F1 dark-state structure in Fig. 5F), while the kinked, highly asymmetric YF1 structure better covers the dark-state envelope (Fig. 5E).

In conclusion, the SAXS data recorded for SB2F1 in the dark and light states reveal substantially larger structural changes than those observed in our crystal structures. These findings support a model of YF1/SB2F1 photoactivation that involves unkinking of the DHp spine, with a light-driven transition from an asymmetric, kinked dimer in the dark to a symmetric, straight dimer in the light.

### Dark-state DHp kinking is enforced by steric clashes at the Jα dimer interface

The above data and photoactivation model therefore prompt the question which structural features give rise to the kinked conformation in the dark state and, conversely, how illumination promotes unkinking of the DHp coiled coil.

**Fig. 6.**
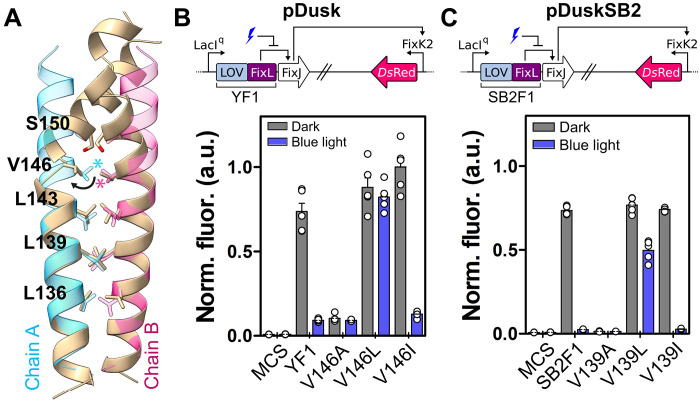
Functional analysis of steric constraints mediating dark-state kinking in YF1/SB2F1. (**A**) Structural model of a canonical (straight) YF1 Jα coiled coil, build using CCBuilder 2.0 (*111*), with individual chains (shown as cyan and pink transparent cartoon) superimposed on the kinked YF1 Jα coiled coil. Residues of the hydrophobic core are shown in stick representation. The black arrow marks the potential displacement of V146 induced by steric clashes in the canonical coiled-coil structure. The pink and cyan asterisk mark the position of potential clashes between the opposing V146 side chains in the canonical coiled-coil model. (**B** and **C**) Functional analysis of YF1-V146/SB2F1-V139 mutations. For the analysis of the functional consequences mutations, a reporter gene assay based on the previously described pDusk and reporter construct (*39*) and the here newly established pDuskSB2 reported (based on SB2F1) [top part in (B) and (C)] was used. In the pDusk/pDuskSB2 constructs, the LacI^q^ promoter controls the expression of the genes encoding for the SHKs YF1/SB2F1 and the RR FixJ from *B. japonicum*. In the dark, the SHK drives FixJ phosphorylation, activating the expression of the reporter gene encoding for the fluorescence reporter *Ds*Red from the FixK2 promoter. Bottom panels: Bacteria harboring the pDusk reporter plasmid containing parental YF1/SB2F1 as well as the YF1-V146 and corresponding SB2F1-V139 variants were incubated under blue light (blue bars) or in the dark (gray bars). *Ds*Red fluorescence of all variants was determined from five biologically independent replicates (depicted as means ± SD). As negative control a construct lacking the fluorescent protein but containing a multiple-cloning site (MCS) was used.

To investigate the structural basis of the kink, we generated a structural model of a canonical (straight) YF1 Jα coiled coil. The individual chains of the model were superimposed onto the first two heptad repeats of the YF1 Jα structure (Fig. 6A). Clash analysis of the canonical model identified V146 as a primary site, where steric clashes between opposing chains could destabilize the coiled coil [Fig. 6A (marked by asterisks) and fig. S9], potentially initiating kinking. When we compare the experimentally determined, kinked YF1 Jα coiled coil to the modeled, straight one, we observe that in the kinked structure V146 is moved out of the interface (Fig. 6A, black arrow).

To probe the role of V146 in mediating kinking (thereby controlling SHK kinase/phosphatase activity), we introduced the V146A, V146I, and V146L mutations in YF1 in the pDusk reporter system (*39*) and measured *Ds*Red reporter fluorescence in darkness and under blue light illumination (Fig. 6B). Mutation to a smaller hydrophobic residue such as alanine is expected to relieve steric constraints at this position, thereby avoiding V146-mediated clashing within the coiled coil. As a result, this substitution likely stabilizes a straight conformation corresponding to the kinase OFF state, independent of photoactivation, which is consistent with our experimental observations. In contrast, substitution with the bulkier leucine side chain (V146L) is predicted to introduce steric hindrance that promotes kinking of the coiled coil, thus favoring the kinase ON state irrespective of illumination. The substitution of V146 with the structurally similar β-branched isoleucine (V146I) apparently only exerts minimal structural and functional perturbation and has only minor effects on activity. Together, these data support a model in which residue size at position 146 critically determines the balance between straight and kinked conformations of the SHK coiled coil, thereby modulating its light-dependent signaling behavior. To ascertain if the same model also applies to SB2F1, we generated a derivative of the pDusk reporter construct, containing SB2F1 instead of the YF1 (pDuskSB2). Using this newly generated optogenetic tool, we assessed the impact of mutating residue V139 (corresponding to V146 in YF1) by measuring *Ds*Red reporter fluorescence in darkness and under blue light illumination (Fig. 6C). For the parental pDuskSB2 construct, a high (28-fold) difference between dark- and light-grown *E. coli* cells can be seen, suggesting that pDuskSB2 represents an effective tool for controlling gene expression in *E. coli* by light. Furthermore, the V139A, V139L and V139I variants yielded very similar results in the reporter assay as in the YF1 context, further suggesting that the mode of signal relay is conserved between YF1 and SB2F1.

## DISCUSSION

### Structural analyses hint at intrinsic conformational equilibria

**Fig. 7.**
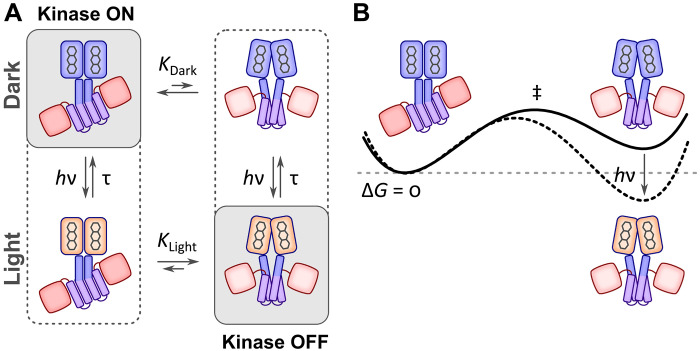
Conformational equilibria and signaling model of SB2F1. (**A**) Kinetic model of YF1/SB2F1 activation. In both the dark and the light state, the YF1/SB2F1 SHK is in dynamic equilibrium between a kinase ON and kinase OFF state. Illumination (*h*υ) changes the equilibrium between the two states from *K*_Dark_ in the dark to *K*_Light_ and thereby modulates kinase activity. In terms of structure, this transition might be accompanied by the change from an asymmetric kinase ON to a symmetric kinase OFF dimer arrangement. The predominately populated states in darkness and under illumination are highlighted in gray. (**B**) Thermodynamic model of SB2F1 activation. Black and dashed lines denote the free-energy surfaces of the dark state and the light state, respectively. The kinase ON state shown in the left has been arbitrarily assigned a Gibbs free energy of zero.

Irrespective of illumination, the arrangement of the LOV-LOV dimers in all our SB2F1 structures more closely resembles the light-state conformation of short-LOV protein structures than the dark-state conformation (Fig. 3, C and D; table S2; and fig. S5) (*45*, *47*, *68*). Strikingly, exposure to light only induced overall modest structural changes within the SB2F1 crystal structures. By contrast, small-angle x-ray solution scattering revealed significantly larger structural changes induced by light (Fig. 5). Whereas the solution scattering data acquired in the light agreed well with the SB2F1 structures, those in the dark did not but were instead consistent with the YF1 structure that shares with SB2F1 the identical DHp-CA module. This conundrum can be rationalized by assuming that LOV photoreceptors exist in a conformational equilibrium between light and dark states. These intrinsic equilibria widely underlie function and allostery of many proteins (*69*, *70*), not least in the model LOV2 domain of *Avena sativa* phototropin-1 (*As*LOV2), as revealed by nuclear magnetic resonance spectroscopy (*71*, *72*). Likewise, HKs dynamically traverse their kinase-active and phosphatase-active states (*22*, *24*). We thus posit that these equilibria also govern the SHKs studied at present and affect crystallization (Fig. 7). Certain crystal lattices may thereby promote the adoption of a dark state–like conformation, while other conditions select for a light state–like conformation. Consistent with this notion, the SB2F1-I66R dark- and light-state crystals all have identical space groups (P3_1_21; trigonal), closely similar unit-cell parameters (table S1) and the same crystal packing (fig. S10), although they were grown in very different conditions. By contrast, YF1 crystallized in a hexagonal P6_5_22 space group and exhibited a pronounced kink in its structure (Fig. 1) (*33*), with its LOV-LOV dimer displaying a short LOV dark state–like dimer arrangement (fig. S11 and table S2).

Functional assays revealed similar enzymatic activities and light responses in SB2F1 and YF1 (Fig. 2 and 6, B and C). In darkness, they acted as net kinases and promoted RR phosphorylation, and while upon exposure to blue light, they switched to net phosphatase activity. On the basis of these findings, SB2F1 and YF1, which share the same HK output module, may use closely similar structural mechanisms for signal transduction. We therefore propose a light-induced transition from an asymmetric/kinked state (embodied by the YF1 dark-state structure) with net kinase activity (kinase ON) to a symmetric/straight state (exemplified by the SB2F1 structures) with net phosphatase activity (kinase OFF) (Fig. 7). Support for this notion derives from SAXS (Fig. 5), functional assays (Fig. 2), similar light state–like LOV-LOV dimer arrangements and overall structure (figs. S12 and S13), and a consistently large H154^…^ATP-γ-P distance (>19 Å) (not allowing for phosphor transfer) in the straight SB2F1 structures.

### Light-dependent changes in Jα coiled-coil interactions drive asymmetry/symmetry transitions modulating kinase/phosphatase activity

**Fig. 8.**
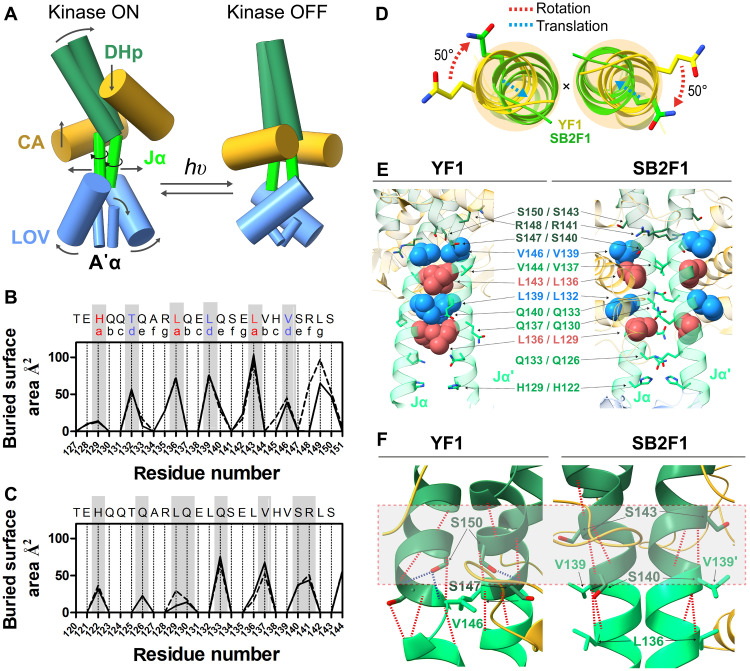
Interface analyses of SB2F1 and YF1. (**A**) Modular representation of asymmetric/kinked (YF1) and symmetric/straight (SB2F1) structures, representing SHK dark (kinase ON) and light states (kinase OFF), respectively. Structural models generated by representing A′α helices, LOV core domains, Jα helices, DHP spine and CA domains by axes for which crossing angles were determined using ChimeraX. (**B** and **C**) Per-residue buried surface area derived from PISA (*79*) analysis of the YF1 (residues 127 to 150) (B) (*33*) and the SB2F1 dark-state Jα helix (residues 120 to 143) (C). Solid and dashed lines denote data obtained for the two chains of the dimer, respectively. Above (B), the Jα helix heptad register identified based on sequence and structure is given. (**D**) Rotational displacement of the Jα coiled coil viewed along the C2 axis (marked by a cross) from the LOV dimer toward the DHp spine. Helix rotation (red dashed line) and translation (cyan dashed line) inferred from LSQKAB superposition of the YF1 (yellow) and SB2F1 Jα helices (green). (**E**) Comparison of Jα-Jα′ coiled coils with hydrophobic core residues as spheres, colored by heptad position (a: red, d: blue). Additional interface residues are shown as sticks. (**F**) Jα-DHp junction in kinked (YF1) and straight (SB2F1) conformations. Key residues, including S150 (YF1) and S143 (SB2F1), highlighted in a gray box. H bonds (<3.2 Å) shown as dashed lines (red: backbone; blue: side chain). In all panels, atom coloring as follows: C in domain/cartoon coloring, O in red, N in blue, and P in orange.

We next analyzed global domain arrangements and local domain interfaces in the asymmetric/kinked YF1 versus the symmetric/straight SB2F1 structures, as representatives of dark and light states, respectively. To this end, we first evaluated the relative orientation of the two LOV protomers within the homodimeric HKs (Fig. 8A). For YF1, we observe a LOV-LOV crossing angle of around 84° compared to about 74° in SB2F1. Likewise, a marked difference between the A′α crossing angles of YF1 (32°) and SB2F1 (78°C) is observed, following the overall trend demonstrated for short LOV proteins (*47*, *68*). The rotational LOV-LOV dimer displacement goes along with a larger separation of the C-termini of the two LOV domains by about 2.1 Å in SB2F1 versus YF1, highly reminiscent of the light-induced reorientation of the LOV-LOV dimer within the PpSB1-LOV protein and of light-induced structural transitions observed in solution for YF1 (*73*, *74*). Notably, the LOV domains rigidly connect to the adjacent Jα helices via their DIT/DVT motifs, a structurally conserved epitope at the C terminus of prokaryotic PAS domains (*75*). Both in the SB2F1 and YF1 structures, the pertinent residues (D118-V119-T120 in SB2F1 and D125-I126-T127 in YF1) engage in several H bonds between each other and a conserved tryptophan in the LOV domain (W103, YF1; W94, SB2F1), thereby firmly anchoring the Jα helices to the LOV core (fig. S14). In addition, residues at the intersubunit interface, previously noted to be important for signaling in PpSB1-LOV/PpSB2-LOV (*56*), such as E96 and K117, contribute to the observed mode of LOV-LOV dimer formation (fig. S14). Thus, LOV domain rotation and slight pivoting apart of the DVT/DIT attachment points directly feed into the Jα helices and affect their relative orientation.

In YF1, said Jα helices were proposed to form a short coiled coil, arguably providing a rigid conduit along which the light signal propagates to the HK effector module (*33*). Consistent with this model, two independent sequence-based coiled-coil prediction tools (*76*, *77*) suggest the presence of a coiled-coil segment covering the Jα helix, in addition to two more within the DHp spine (fig. S15A). The asymmetric YF1 structure indeed exhibits such a parallel Jα coiled coil (fig. S15B) comprising two canonical heptad repeats (*abcdefg*) extending from H129-L149 (corresponding to H122-L142 in SB2F1), with the hydrophobic core formed by L136, L139, L143, and V146 within the *a* and *d* registers (Fig. 8B). The same coiled-coil element, with the same register, is also identified by the structure-based coiled-coil identification tool, Socket2 (*78*), and is also apparent in the repeating pattern of buried residues (*79*) inferred by PISA analysis (Fig. 8B). By contrast, in the symmetric SB2F1 structure, the LOV protomers pull the Jα helices apart at their N-terminal ends and thereby preclude the formation of a canonical coiled coil. Notably, Socket2, even under the most relaxed packing cutoff, does not identify a coiled coil in the SB2F1 structure. As a corollary, the N-terminal portions of the Jα helices of SB2F1 run in parallel rather than being wound around another as in the YF1 structure. Intriguingly, the C-terminal halves of the Jα helices in the SB2F1 structure are sufficiently close to assemble into α-helical bundle, albeit with the heptad register shifted by one residue compared to the YF1 structure (identified by the repeating pattern of buried residues, Fig. 8C). For instance, residue V144 occupies the coiled-coil register *b* in the YF1 structure, but the corresponding residue V137 in the SB2F1 structure falls within the register *a*. The altered register principally owes to a 50° rotation of each of the two Jα helices making up the coiled coil that amounts to a 100° relative reorientation of individual residue pairs within the juxtaposed helices (Fig. 8D).

As a consequence of this rotation, the hydrophobic core of the N-terminal segment of the Jα helix becomes partly solvent exposed in the SB2F1 structure (Fig. 8, E) which contributes to a much reduced buried surface area and less stable Jα-Jα′ interface [solvation energy gain on complex formation of the SB2F1 Jα-DHp interface of ~−19.3 kcal/mol compared to ~−24.9 kcal/mol for YF1 (*79*)]. Given the heptad periodicity of parallel dimeric coiled coils, consecutive residues within the constituent helices are angularly distributed in 100° increments around the helix spine. The differences in Jα helix orientation between the YF1 and SB2F1 and their impact on coiled-coil packing can thus be deemed roughly equivalent to insertion/deletion of single residues within the coiled-coil helices would have (also seen from the +1-residue shift of buried residues identified by interface analysis; Fig. 8C). Ample biochemical data on YF1 and derivative variants indeed show that insertion of single residues within the Jα helix can lead to inversion of the response to light (*80*). Collectively, these findings suggest that light promotes Jα helix rotation and alters coiled-coil packing interactions, which likely translate into structural rearrangements in the DHp and CA domains, ultimately linked to catalysis (see below).

Comparative structural analyses (Fig. 8, D and E) together with mutational data (Fig. 6) suggest that DHp kinking is a direct consequence of Jα helix rotation during the light-dark transition. In the light state, the Jα helices adopt a symmetric, straight two-helix bundle, incompatible with a canonical coiled-coil knobs-into-holes packing. Transition to the dark state involves rotational rearrangements of the helices, which repositions residues at the dimer interface to enable coiled-coil packing interactions (Fig. 8E). During the transition, the bulky side chains of V146/V139 become sterically incompatible with the canonical packing geometry (Fig. 6A) generating clashes between opposing helices. We propose that these steric constraints are relieved by local deformation of the coiled coil, resulting in the kinked DHp conformation observed in the dark-state structure (Fig. 8A). Consistent with this mechanism, reducing side-chain volume at position 146/139 of YF1/SB2F1 promotes the phosphatase-active state, whereas increasing steric bulk effectively locks the receptor in its kinase-active state. Along similar lines, the random mutagenesis of the Jα coiled coil in YF1 also identified several additional positions, including Q133, R135, E142, and L143, for which substitution yield constitutively active (ON) variants (*81*), while site-directed proline substitutions at L136 and L139 result in constitutively low kinase (OFF) activity (*33*). This indicates that the sensitivity to mutation is not confined to V146 but extends along the Jα helix.

As a consequence of the kinking/unkinking transition, in the kinked YF1 structure, the downstream S150 side chain is rotated to face into the hinge region, forming H-bonding contacts to the backbone of S147 and V146 (Fig. 8F), thereby stabilizing the kink. In turn “unkinking” or straightening of the DHp spine, as seen in the straight SB2F1 structure, is a consequence of rational (torque-like) movement of the Jα coiled coil, which extends beyond the Jα element, with S150 (S143 in SB2F1) and V146 (V139 in SB2F1) being rotated out of the coiled-coil interface (Fig. 8F). In turn, this results in altered hydrophobic and polar Jα-DHp-CA domain contacts, which might contribute to the stabilization of the symmetric/straight structure (figs. S16 and S17).

Following this model, the question remains, how these structural transitions are linked to regulating kinase/phosphatase activity? As outlined in Results section, in the symmetric/straight dimer, the distance between the phospho-accepting H154 (corresponding to H161 in YF1) and the ATP γ-phosphate (Fig. 3A) is about 19 to 20 Å, which is too large to allow autophosphorylation, thus supporting the assignment of the symmetric/straight structure to the kinase OFF state. Note, however, that even the asymmetric/kinked YF1 structure does not fully capture the structure of the active kinase ON state, as the distance between the ATP molecule in the CA domain and the phospho-accepting H161 (corresponding to H154 in SB2F1) is with 12.8 Å still too large. Thus, additional conformational changes and/or a certain degree of flexibility are required to enable H161 autophosphorylation. We note that the CA domain connects to the DHp moiety via a linker of around 10 residues length that stands to grant exactly the type of motional freedom required to bring the γ-phosphate of the ATP into spatial proximity of the active-site histidine. However, further studies that directly address intrinsic protein dynamics (*82*, *83*) are needed to understand the importance of SHK flexibility and protein dynamics for catalysis.

In summary, we propose the following general model for photoactivation and signaling in SB2F1 and YF1 (Fig. 8A). In the dark, SB2F1 and YF1 adopt an asymmetric/kinked dimer (similar to the YF1 dark-state dimer; as supported by our SAXS data). Such a conformation, combined with a general flexibility of the CA domains, allows autophosphorylation and phosphotransfer to FixJ (kinase ON state). Illumination triggers FMN-Cys adduct formation and results in a rotational movement of the LOV domains of the LOV-LOV dimer relative to each other. The resulting pivoting apart of the N-terminal ends of the Jα helix is accompanied by a change of the overall Jα crossing angle and a torque-like rotational movement that translates into the subsequent DHp helical spine, resulting in a straight, more symmetric HK module conformation, with the phospho-accepting histidine moving away from the CA domain (kinase OFF state). Such a change is supported by our SAXS data and by the observation of light state–like LOV-LOV dimer arrangements seen for all of the here determined structures.

### Dimer asymmetry and flexibility drive signal transduction in LOV-SHKs and beyond

Apart from being relevant for designed SHKs such as YF1 and SB2F1, the suggested signaling mechanism might also be realized by natural (LOV)–SHKs. DHp domain dimer asymmetry has long been associated with SHK signaling. One prevailing model suggests that SHKs exist as symmetric dimers with relatively straight α helices in the DHp subdomain in the inactive/phosphatase state (kinase OFF state) (*84*). Upon activation (kinase ON state), the dimer becomes asymmetric: One subunit adopts an autophosphorylating conformation, while the other engages in phosphotransfer with the RR. This transition involves bending of the DHp helices to accommodate the asymmetry. Hence, the kinase ON state is associated with DHp bending/dimer asymmetry (*84*), while the kinase OFF state is associated with a symmetric/straight dimer structure. A similar model has been suggested for the natural LOV-HK of *Brucella abortus*, which comprises an N-terminal LOV domain, a PAS domain, and a C-terminal HK module (DHp and CA domains) (*11*, *34*). Crystal structures revealed that the dark-state (kinase OFF) LOV-PAS dimer (lacking the HK module) is straight and symmetric, while the light-state (kinase ON) full-length LOV-PAS-HK dimer is highly asymmetric and kinked (*34*). Solution scattering experiments and photoexcitation studies of the dark-grown LOV-PAS crystals further suggested a light-induced transition from a straight (kinase OFF) to a kinked asymmetric dimer (kinase ON) (*34*).

In addition, the suggested mode of signal-relay by rotational movement of a marginally stable coiled coil shares similarities with the signaling mechanism proposed for the periplasmic SHK BvgS of the whooping cough agent *Bordetella pertussis* (*85*), which together with its cognate RR BvgA regulates the expression of virulence factors necessary for infection. Each monomer of dimeric BvgS consists of two tandem periplasmic Venus fly trap domains, a transmembrane segment and a cytoplasmic PAS domain, linked via a helical connector that forms a parallel coiled coil in the dimer to a HK output module. Intriguingly, in BvgS and related proteins, a DIT/DVT motif links the PAS domain to the coiled-coil PAS-HK connector. On the basis of biochemical and reporter gene experiments, it was shown that a flexible, rotationally dynamic, coiled coil favors the kinase ON state, while in the phosphatase-active (kinase OFF) state, the coiled-coil adopts a more rigid conformation that buries its hydrophobic interface (*85*). This feature, although in reverse, is highly reminiscent of the model we suggest for YF1/SB2F1 signaling.

In conclusion, our study provides insights into the signaling mechanism of engineered light-dependent LOV-HKs, highlighting that, similar to other LOV photoreceptors, they exist in a dynamic conformational equilibrium between light and dark states. This equilibrium, together with stabilizing interdomain and crystal contacts, might influence the structural outcomes observed in crystallization experiments, underscoring the importance of careful interpretation when assigning functional states based on full-length SHK structures. This is reminiscent of the discussion evolving for phytochrome-based TCSs, where structures of these proteins have yielded a number of partially conflicting conformational mechanisms for signal transduction [see (*86*) and discussion and references therein]. The internal flexibility of the HK module (comprising the DHp and CA domains) reflects a key feature necessary for SHK function. This structural adaptability complicates crystallization but plays a crucial role in signaling and may lead to variations in observed conformations. Our data corroborate the central role of dimer asymmetry and HK module flexibility in SHK signaling, underscoring the importance of conformational diversity and dynamics in SHK function. As photoreceptor HKs serve as broadly applicable models for SHKs, our findings likely extend to many other members of this protein family, providing a framework for interpreting structural data and guiding the design of novel light-sensitive signaling systems for optogenetics, as exemplified here for the newly derived SHK SB2F1.

## MATERIALS AND METHODS

### Cloning and site-directed mutagenesis of SB1F1, SB2F1, and SB2F1-I66R

The SB1F1 and SB2F1 fusion constructs were generated by overlap-extension polymerase chain reaction (PCR) (*87*, *88*). In brief, the gene fragments encoding for the N-terminal end of the PpSB1-LOV and PpSB2-LOV photoreceptors (residues 1 to 120; comprising the A′α helix and the LOV core domain; Fig. 1B) were amplified using the oligonucleotides SB1_ov_fw (5′-CGCGGCAGCCATATGATCAACGCGCAATTGC-3′)/SB2_ov_fw (5′-GGCGGCCATATGATCAACGCAAAACTCCTGCAACTGATGG-3′) and SB1_ov_rev (5′-GTCTGCTGGTGCTCGCTGACGTCCTTCTGG-3′)/SB2_ov_rev (5′-GCCTGGGTCTGCTGGTGCTCTGTGACATCGCGCTGGATGC-3′) using a pET28a + vector carrying the PpSB1-LOV/PpSB2-LOV–encoding genes (*52*) as template. The gene fragment encoding the HK domain of FixL (amino acids 128 to 275 of YF1; including Jα helix portion of the DHp domain; Fig. 1B) was amplified using the oligonucleotides FixL_ov_fw (5′-GAGCACCAGCAGACCCAGGCGCGTCTCCAG-3′) and FixL_ov_rev (5′-GCGGCCGCAAGCTTGTCGACTCAATTCTCGTC-3′) with pDusk (*39*) as template. The two gene fragments were fused in a subsequent overlap-extension PCR using the oligonucleotides SB1_ov_fw/SB2_ov_fw and FixL_ov_rev. The resulting PCR product was hydrolyzed with NdeI and SalI and ligated into an identically digested pET28a + vector (Novagen-Merck, Darmstadt, Germany). This cloning strategy enables the expression of the gene fusion in suitable *E. coli* expression strain under the control of the strong P*_T7_* promoter and simultaneously results in the fusion of an N-terminally encoded hexa-histidine tag (His_6_-tag; tag sequence: MGSSHHHHHHSSGLVPRGSH), which allows easy purification of the fusion protein via immobilized metal ion affinity chromatography (IMAC). SB2F1-I66R was generated by QuikChange PCR using the oligonucleotides SB2F1-I66R_fw (5′-GGATCACGACCAGCCGGGCCGTGCAATTATCCG-3′) and SB2F1-I66R_rev (5′-CGGATAATTGCACGGCCCGGCTGGTCGTGATCC-3′) and the pET28a + vector carrying the SB2F1 encoding gene fusion (see above) as template. Likewise, the V146A, V146I, and V146L mutations in YF1 within the pDusk construct (*39*) were introduced via the QuikChange PCR (Agilent Technologies) using the oligonucleotides: D16_V146A_fwd (5′-GCTCGTCCACGCCTCCAGGCTGAGC-3′), D11_V146A_rev (5′-GCTCAGCCTGGAGGCGTGGACGAGC-3′), D12_V146I_fwd (5′-GCTCGTCCACATCTCCAGGCTGAGC-3′), D13_V146I_rev (5′-GCTCAGCCTGGAGATGTGGACGAGC-3′), D14_V146L_fwd (5′-GCTCGTCCACCTCTCCAGGCTGAGC-3′), and D15_V146L_rev (5′-GCTCAGCCTGGAGAGGTGGACGAGC-3′). All constructs were verified by sequencing (Microsynth-SeqLab GmbH, Göttingen, Germany). In addition, residue D55 of FixJ was exchanged to alanine within the background of a pET19-SUMO expression construct (*55*) (forward: 5′-CGTCGTCTCCGCCGTGCGCATGC-3′; reverse: 5′-GCATGCGCACGGCGGAGACGACG-3′).

### Generation of the SB2F1-based reporter construct pDuskSB2

The gene fragment encoding the PpSB2-LOV domain was amplified by PCR from the SB2F1 expression construct and used to replace the fragment encoding the *B. subtilis* YtvA-LOV domain with a pDusk-*Ds*Red reporter plasmid (*39*). Cloning was done by Gibson assembly using the following primers for the amplification of insert and backbone: pDusk-SB2 (forward: 5′-CAATTGAGGGTGGTGAATGTGATCAACGCAAAACTCCTGCAACTGATGG-3′; reverse: 5′-GCAGGAGTTTTGCGTTGATCACATTCACCACCCTCAATTGACTCTCTTCC-3′) and SB2-pDusk (5′-GCATCCAGCGCGATGTCACAGAGCACCAGCAGACCCAGGCG-3′; reverse: 5′-CTGGGTCTGCTGGTGCTCTGTGACATCGCGCTGGATGCCGATG-3′). Residue substitutions at position V139 (corresponding to V146 in YF1) were obtained in a similar fashion by amplifying PCR fragments from the above pDusk vectors.

### Heterologous gene expression

For characterization purposes, each of the above-mentioned constructs having an N-terminal His_6_-tag) was expressed in *E. coli* BL21(DE3), as described previously for the corresponding short LOV proteins (*45*, *47*, *53*). Autoinduction (AI) media (*89*) was prepared with terrific broth medium (X972; Carl-Roth, Karlsruhe, Germany) and supplemented with 50 μM riboflavin (A6279; AppliChem GmbH, Darmstadt, Germany), kanamycin (50 mg/liter), and, for induction, glucose (0.5 g/liter) and lactose (20 g/liter). Overexpression was carried out in 250-ml AI media cultures for 3 hours at 37°C with continuous shaking at 110 rpm. Afterward, the incubation temperature was reduced to 30°C, and the cells were incubated under constant agitation for another 24 hours.

YF1 and FixJ/FixJ-D55A were expressed in *E. coli* CmpX13 (*90*) as described before (*33*, *55*). LB medium (1 liter) was inoculated with a 5-ml starter culture and grown at 37°C and 225 rpm to an optical density at 600 nm (OD_600nm_) of 0.6. For YF1 and FixJ/FixJ-D55A, the medium was supplemented with kanamycin (50 μg/ml), 50 μM riboflavin, or ampicillin (50 μg/ml), respectively. Expression was induced by adding 1 mM isopropyl-β-d-thiogalactopyranoside (IPTG), and cultures were incubated overnight at 16°C and 225 rpm.

#### SeMet labeling

The selenomethionine (SeMet) labeling protocol was adapted from Doublié (2007) (*91*) using the methionine auxotrophic *E. coli* strain B834 (DE3) (Novagen, Darmstadt, Germany) and SeMet medium (*91*) for growth. To enable adaptation to the SeMet medium, the cells were resuspended at an OD_600nm_ of 0.1 in 1-liter SeMet medium supplemented with l-methionine at a concentration of 50 mg/liter (500 ml of culture in 2-liter baffled Erlenmeyer flasks) and grown at 37°C with shaking at 130 rpm until an OD_600nm_ of 1.0 to 1.5 was reached. The cells were subsequently centrifuged at 3000*g* for 15 min and resuspended at an OD_600nm_ of 0.35 in SeMet medium and incubated for 6 hours at 37°C and 130 rpm to deplete the remaining l-methionine. Then, l-selenomethionine (purchased from Thermo Fisher Scientific, Waltham, USA) was added to a final concentration of 75 mg/liter and incubated with a gradual temperature reduction to 30°C until an OD_600nm_ of 0.6 was reached. Subsequently, gene expression was induced by addition of 1 mM IPTG. After incubation for another 4 hours, the cells were harvested by centrifugation at 5000*g*. The wet cell pellets were either used directly or frozen at −20°C until further use.

### Protein purification

SB1F1, SB2F1, and SB2F1-I66R were purified by using IMAC as described previously (*45*, *47*, *52*, *53*, *68*). The fractions containing purified protein obtained from IMAC were pooled, and the imidazole-containing elution buffer was exchanged with the storage buffer [20 mm tris (pH 8.0) and 40 mm NaCl] using an ÄKTA pure FPLC system (GE Healthcare, Buckinghamshire, UK) with a HiPrep 26/10 Desalting column as per standard protocol. The eluted protein fractions were pooled supplemented with 4 mM tris(2-carboxyethyl)phosphine (TCEP) and concentrated by ultrafiltration using Vivaspin centrifugal concentrator units (molecular mass cutoff: 10 kDa) (Sigma-Aldrich, St. Louis, MO, USA). SEC was used as the last step in purification and for molecular weight estimation. Superdex 200 10/300 GL and Superdex 200 Increase 10/300 GL columns (GE Healthcare, Buckinghamshire, UK) were used with an ÄKTA pure FPLC system. The standard running buffer was 20 mM tris at pH 8.0 and 40 mM NaCl. The flow rate was set to 0.5 ml/min.

YF1 was purified as described before (*33*). In brief, the harvested cells were disrupted by sonification, and the His-tagged YF1 was isolated via IMAC. Fractions were pooled according to yield and purity, and samples were stored in 20 mM tris-HCl, 20 mM NaCl, and 10% (v/v) glycerol (pH 8.0).

FixJ and FixJ-D55A was purified in two steps as described before (*55*). After cell harvesting and disruption by sonification, the His_6_-SUMO–tagged FixJ/FixJ-D55A was first isolated via IMAC. Fractions were pooled and the SUMO-tag was cleaved off in 50 mM tris and 1 M NaCl (pH 8.0) overnight at 4°C by His-tagged Senp2 protease. Second, in a reverse IMAC, FixJ/FixJ-D55A was collected in the flow-through, concentrated, and stored in 20 mM tris-HCl, 250 mM NaCl, and 10% (v/v) glycerol (pH 8.0).

### Determination of chromophore content and loading

The quantification of chromophore loading and content was performed by UV-Vis spectroscopy and high-performance liquid chromatography as described previously (*52*, *53*).

### Dynamic light scattering

DLS measurements were performed using a SpectroSize 300 instrument (Xtal Concepts, Hamburg, Germany) at 20°C. Approximately 50 μl of protein solution (~1 mg/ml) in the storage buffer described above was used for each measurement. Before analysis, all samples were centrifuged at 14,000*g* for 10 min. Scattering was recorded at a wavelength of 660 nm and an angle of 90° using a quartz glass cuvette. Measurement time was 10 s, with 20 acquisitions collected per sample. Diffusion coefficients were determined by analyzing the decay of the scattered intensity autocorrelation function, and hydrodynamic radii were calculated using the SpectroCrystal software provided by the manufacturer.

### Spectroscopic techniques

UV-Vis spectrophotometric measurements were carried out with a Shimadzu UV-1800 spectrometer (Shimadzu, Kyoto, Japan) as described previously (*45*, *47*, *52*, *53*, *92*). The samples were illuminated for at least 30 s using a blue light (λ_max_ = 450 nm) emitting light-emitting diode (LED) with a radiant power of 50 mW (Luxeon Lumileds, Phillips, Aachen, Germany) to generate the respective light states. Dark recovery kinetics were then measured from the illuminated samples as described previously (*45*, *47*, *52*, *53*, *92*). The absorbance recovery at 475 nm was recorded as a function of time. The absorbance data were plotted against time with the program Origin and fitted using a single exponential decay function. All measurements were done in triplicate.

### Functional characterization of SB1F1, SB2F1, and SB2F1-I66R

The net HK activities of the SB1F1, SB2F1, and SB2F1-I66R proteins were assessed via a fluorescence anisotropy assay as reported before (*55*). The binding site of phospho-FixJ (underlined) was included in TAMRA-labeled, double-stranded DNA (5′-GAGCGATATCTTAAGGGGGGTGCCTTACGTAGAACCC-3′), which was prepared as described previously (*93*). Samples were prepared under red safe light. Enzymatic measurements were conducted in 20 mM tris-HCl, 80 mM NaCl, 2.5 mM MgCl_2_ (pH 8.0) with 2.52 μM protein variant, 25.2 μM FixJ, 1.26 μM TAMRA-dsDNA, and bovine serum albumin (111 μg/ml) (*38*). Samples were transferred into a black 96-well microtiter plate (FluoroNunc) and equilibrated at 25°C for 5 min. The kinase reaction was started by adding 1 mM ATP, and the TAMRA fluorescence anisotropy was monitored with a microtiter plate reader (CLARIOstar, BMG Labtech) at (540 ± 20) nm excitation and (590 ± 20) nm emission. After 25 min, the sample was ejected and illuminated with 450-nm light for 15 s before continuing the measurement. To validate the origin of the fluorescence anisotropy change, control reactions were conducted with a FixJ variant in which the phospho-accepting D55 was replaced by alanine (FixJ-D55A) and with a scrambled TAMRA-labeled DNA substrate of the same length and GC content but shuffled sequence (5′-GGGCAGTACTGCGTGGATTATGGACGTAATACCCCAG-3′; prepared as described above) (fig. S18).

### Functional analysis of YF1/SB2F1 mutations

The YF1 and SB2F1 variants were analyzed on the basis of the expression of a *Ds*Red reporter in the pDusk and pDuskSB2 contexts (*39*). *E. coli* CmpX13 (*90*) containing the original YF1, SB2F1, or the respective mutations were used to inoculate 200-μl LB medium supplemented with kanamycin (50 μg ml^−1^; LB-Kan) in a black-walled microtiter plate (MTP) with clear bottom (μClear, Greiner BioOne, Frickenhausen, Germany) in triplicates. As background control an empty plasmid variant with a multiple-cloning site was used. MTPs were sealed with a gas-permeable membrane, and bacteria were incubated for 21 to 24 hours at 30°C and 750-rpm agitation in blue light. Illumination was applied constantly via an Arduino-controlled setup with a LED peak wavelength of (463 ± 12) nm and an intensity of 60 μW cm^−2^ (*94*). After diluting a 100-fold in fresh LB-Kan medium under red safe light, the cultures were split into two. A 200 μl was incubated in a clear MTP in darkness, and the other 200 μl was in a black-walled MTP with clear bottom in blue light as described before. After 18 hours at 37°C and 750-rpm agitation, the optical density at (600 ± 9) nm (OD_600_) and the *Ds*Red fluorescence [(554 ± 9) nm excitation and (591 ± 20) nm emission] were measured with a Tecan Infinite M200 Pro MTP reader (Tecan Group Ltd., Männedorf, Switzerland). Data represent the means ± SD of three biological replicates, and the *Ds*Red fluorescence was normalized by OD_600_.

### Protein crystallization

The purified proteins were concentrated to ~4 mg/mL. Crystallization experiments were performed using the vapor diffusion method in 96-well sitting-drop plates, with a drop size of 1.8 μl (0.9 μl of purified protein plus 0.9 μl of reservoir solution) against 70 μl of the reservoir solution. Both SB2F1 and SB2F1-I66R proteins were crystallized at 19°C under dark conditions where the plates were kept wrapped in aluminum foil, with the reservoir containing 10 to 12% PEG-8000 (polyethylene glycol, molecular weight 800; v/v), 0.2 M NaCl, 0.1 M sodium citrate (pH 5.8 to 6.1), 1 mM ATP, and 2 mM MgCl_2_. Before cryo-cooling, 20% PEG-200 (v/v) was added in small steps (~5%) to the drop containing crystals. All the steps were performed in the dark or under low red light conditions. SB2F1-I66R was crystallized at 19°C under continuous light conditions where the plates were constantly illuminated with custom-made blue light LED arrays (λ_max_ = 450 nm, Luxeon Lumileds, Phillips, Aachen, Germany), with the reservoir containing 16 to 20% PEG-3350 (v/v), 0.2 M LiSO_4_, 0.1 M bicine (pH 9.0 to 9.3), 1 mM ATP, and 2 mM MgCl_2_. As above, 20% PEG-200 (v/v) was added in small steps (~5%) to the drop containing crystals before cryo-cooling.

### Single-crystal microspectrometry

UV-Vis absorbance spectra of cryo-cooled crystals at 100 K were recorded in the wavelength range of 250 to 700 nm using a microspectrometer at the beamline ID29S at European Synchrotron Radiation Facility (ESRF) (Grenoble, France) as described previously (*95*). The spectra of protein crystals were measured both before and after x-ray exposure during data collection.

### Data collection and structure determination

Single crystals were mounted in loops and flash frozen with gaseous nitrogen at a temperature of 100 K. All steps for data collection on crystals obtained in dark conditions, including data acquisition at the beamline, were performed under low red light conditions. X-ray diffraction data at 100 K were recorded at the ESRF beamlines ID29 (*96*) and ID30B (*97*) at Grenoble, France. The respective wavelengths and corresponding detector types of each beamline are listed in table S1. The strategy for data collection was determined using the BEST program to reduce potential radiation damage from the beam while maintaining data collection as complete as possible (*98*). Data processing was performed using the XDS program (*99*) and AIMLESS [part of the CCP4 package (*100*)]. At first, we tried to obtain the initial phases by molecular replacement using the published YF1 structure [PDB ID: 4GCZ, (*33*)] as a search template. However, this procedure failed despite high sequence similarity (fig. S1) and expected similar state (crystallization under dark conditions in presence of ATP/MgCl_2_). Therefore, to obtain initial phase information, we produced SeMet-labeled protein as mentioned above. SAD data were collected for the crystals obtained using the SeMet-labeled protein (table S1). Moreover, the crystals of the SeMet-labeled protein diffracted to a higher resolution. A comparison of native and SeMet-labeled protein structures showed that the incorporation of the SeMet in place of methionine did not induce any structural changes in the protein. Therefore, all presented crystal structures of SB2F1 and SB2F1-I66R were determined from crystals obtained from SeMet-labeled protein.

For the SB2F1-I66R crystals, the initial phases were determined by molecular replacement using the program MOLREP (CCP4 package) (*100*). The search model was the crystal structure of SB2F1 in the dark state, obtained as part of this work. The models described were further improved with several cycles of refinement using the program PHENIX (*101*) and manual rebuilding using the COOT graphics program (*102*). Data collection and refinement statistics are listed in table S1.

The analysis of the SB2F1 datasets revealed that the majority of crystals belonged to the P3_1_21 space group, with unit cell dimensions of *a* = *b* ≈ 138 Å and *c* ≈ 97 Å (table S1; PDB ID: 8A6X). A smaller subset of datasets (<5%) exhibited crystals in the closely related P3_2_21 space group, characterized by unit cell dimensions of *a* = *b* ≈ 138 Å and *c* ≈ 48 Å (table S1, PDB ID: 8A3U). Notably, both crystal forms originated from the same crystallization drop, indicating that the variation in space group was not due to differences in crystallization conditions. The structural superposition of the two models demonstrated that they are nearly identical, with a Cα RMSD of 1.09 Å over residues 1 to 365 (table S2). Given that all other structures analyzed in this study adopt the P3_1_21 space group, the SB2F1 dark-state structure corresponding to PDB ID: 8A6X is used for subsequent discussion and comparison.

### Small-angle x-ray scattering

SAXS data for all proteins were collected at beamline BM29 at the ESRF (Grenoble, France) (*103*, *104*), as described previously (*50*). The SB2F1 protein solution in buffer [20 mM Hepes (pH 7.5), 150 mM NaCl, 2% glycerol, 2 mM TCEP, 1.5 mM ATP, and 3 mM MgCl_2_] was concentrated using Vivaspin 20 (Sartorius, Göttingen, Germany). The filtrate was collected and used as control during SAXS measurements. The reference measurement of the protein buffer was done before and after each protein sample. For the measurements of the protein dark state, all sample manipulations were performed in the experimental hutch in the dark under red light conditions. For the light-state experiments, the protein solutions were illuminated with a blue light LED (wavelength of 450 nm, radiant power of 50 mW; Luxeon Lumileds, Phillips, Aachen, Germany) continuously in the sample storage position, and the SAXS experiment was performed under standard light conditions. The temperature for data collection was set to 10°C to slow down the dark recovery, thus enabling more efficient population of the light state. The x-ray wavelength used on BM29 was 0.992 Å, and the used protein concentrations were 0.64, 1.45, 2.95, and 4.18 mg/ml. For each sample, 10 frames with an exposure time of 1 s each were recorded. The individual recorded frames were checked for the absence of radiation damage, and the corresponding frames were merged. The scattering contribution of the buffer was subtracted from the merged datasets of the protein solutions. The buffer-subtracted SAXS data were scaled by the measured protein concentrations. Data measured of the above-mentioned protein concentrations were merged for further data analysis. The final datasets used for evaluation were obtained by merging 0.64 mg/ml for the smaller *q* range and 4.18 mg/ml data for the higher scattering vector range.

Data were analyzed and modeled using the programs available within the ATSAS software package (*105*). The distance distribution function *P*(*r*) was determined using the program DATGNOM. In addition, experimental SAXS data were compared to theoretical scattering curves of the crystal structures calculated by the program CRYSOL. Ab initio bead models were generated with the DAMMIN program. In both cases, 50 ab initio models were generated, aligned, and averaged to the most probable model. Additional restraints were imposed to improve, for example, the connectivity and compactness of the model. The crystal structures were then aligned with the averaged model. All models were used for further averaging and filtering by DAMAVER as their normalized spatial discrepancy (NSD) was within the range of mean NSD ± 2σ (SB2F1-Dark: mean NSD = 1.406, σ = 0.324; SB2F2-Light: mean NSD = 1.354, σ = 0.367). The envelope function was determined using the SITUS package (*106*).

### Bioinformatic, structural analyses, modeling, and graphical representation

PDB files were edited using open source Pymol version 2.6.0 (Schrödinger LCC, NY, USA) (*107*) or the UCSF ChimeraX software (*108*). For comparative analyses of the YF1 and SB2F1 structure, we used the SB2F1 dark-state structure. Interface analyses were performed using the LigPlot+ v2.2 software tool (*109*) and the ‘Protein interfaces, surfaces and assemblies’ (PISA) service at the European Bioinformatics Institute (www.ebi.ac.uk/pdbe/prot_int/pistart.html) (*79*). Jα helix rotation/translation was analyzed by using LSQKAB (*110*). The SB2F1 and YF1 structures were superposed with respect to the backbone atom positions within the first two turns of their Jα helices in both subunits (chains A and B). Next, the Jα helices of SB2F1 and YF1 were superposed with regard to the same atoms but of a single chain only (either A or B). From the resultant rotation and translation matrices, a screw rotation axis was calculated using custom Python scripts (*33*). Sequence-based coiled-coil predictions were carried out using the deep learning–based coiled-coil prediction tool CoCoNat (*77*) and DeepCoil2 (*76*). For structure-based coiled-coil identification, the Socket2 webservice (*78*) was used. The structural model of the dimeric canonical Jα coiled coil was generated using the CCBuilder 2.0 webservice (https://pragmaticproteindesign.bio.ed.ac.uk/builder/) (*111*). The individual chains of resulting models were superimposed with the YF1 Jα helices (first two turns, backbone atoms only) and analyzed for clashes using ChimeraX (*108*). Unless otherwise indicated, figures were generated with UCSF ChimeraX software (*108*) developed by the Resource for Biocomputing, Visualization, and Informatics at the University of California, San Francisco, using secondary structure assignments as given by the DSSP program (*112*). UV-Vis spectra and functional assays were plotted and analyzed using the Origin 2020 software (OriginLab Corporation, Northampton, MA, USA).

### Accession numbers

Atomic coordinates and structure factors for the two SB2F1 dark-state and illuminated-state structures were deposited in the PDB (www.rcsb.org) under PDB IDs: 8A6X, 8A3U, and 8A52, respectively. SB2F1-I66R dark and light states were deposited under PDB IDs: 8A7F and 8A7H, respectively.

## Supplementary Materials

### This PDF file includes:

Figs. S1 to S18 Tables S1 to S3 References
