# Supplementary material for: Dimer asymmetry in signaling of blue light sensor histidine kinases
Source: Sci Adv. 2026 Jul 1;12(27):eaed8943. doi: 10.1126/sciadv.aed8943 (PMC13322254; doi:10.1126/sciadv.aed8943)
Supplement: Supplementary file 1 — Figs. S1 to S18 Tables S1 to S3 References [file sciadv.aed8943_sm.pdf]

Science Advances
AAAS

Supplementary Materials for

Dimer asymmetry in signaling of blue light sensor histidine kinases

Vladimir Arinkin *et al.*

Corresponding author: Ulrich Krauss, ulrich.krauss@uni-bayreuth.de, u.krauss@fz-juelich.de;
Renu Batra-Safferling, r.batra-safferling@fz-juelich.de

Sci. Adv. **12**, eaed8943 (2026)
DOI: 10.1126/sciadv.aed8943

This PDF file includes:

Figs. S1 to S18
Tables S1 to S3
References

The Supporting Figures and Tables are listed below in the order in which they appear in the main manuscript.

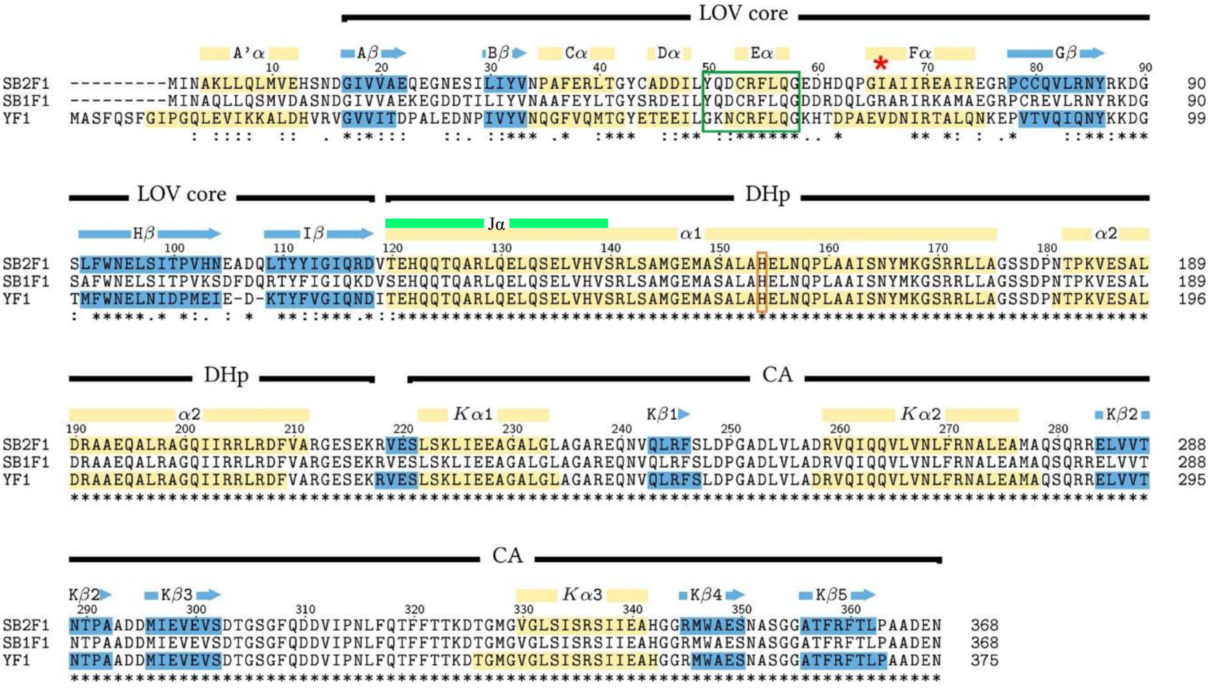

Figure S1: Multiple sequence alignment of SB1F1, SB2F1 and YF1. The dark green box marks the highly conserved GXNCRFLQG motif of LOV domains, which is altered to YXDCRFLQG in PpSB1-LOV, PpSB2-LOV and other *Pseudomonadaceae* short LOV proteins (*68*). The red box marks His154, which is autophosphorylated. The red asterisk highlights the I66 position in SB2F1, which is mutated to Arg in SB2F1-I66R. SHK domain boundaries: LOV-domain, DHp and CA domain are labeled with bars above the alignment. The Jα helix section of the DHP α1 helix, which is coupled to the LOV core via the DIT motif in YF1 (*33*) and the DVT motif in SB1F1/SB2F1, is highlighted in neon green.

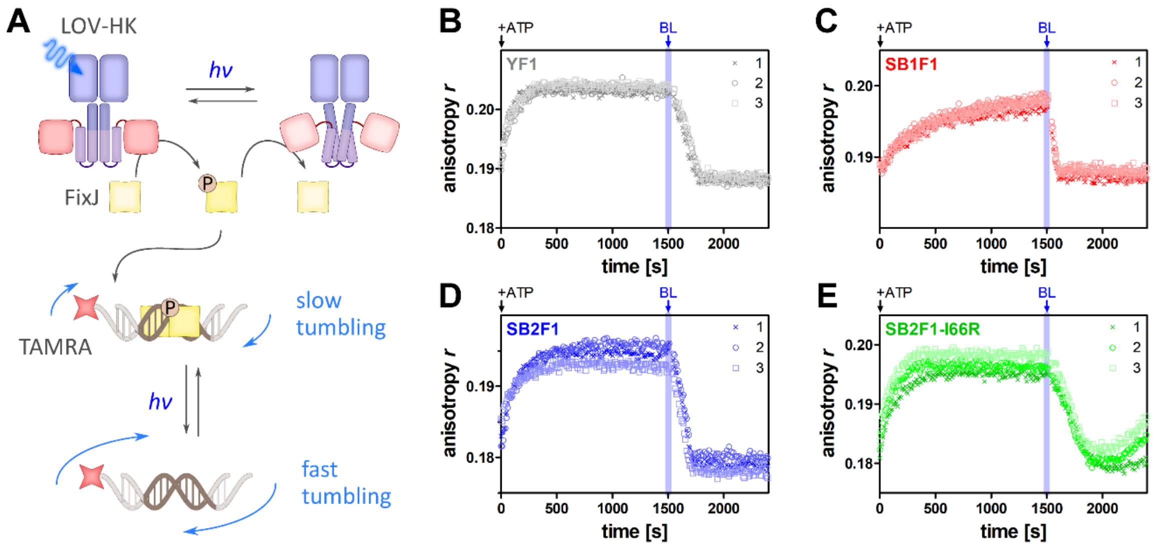

Figure S2: Histidine kinase and phosphatase activity of SB1F1, SB2F1, SB2F1-I66R and YF1 monitored by fluorescence anisotropy. (A) Scheme illustrating the assay principle (*55*). All LOV-HKs possess net kinase activity in the dark phosphorylating the RR FixJ upon addition of ATP. Phospho-FixJ subsequently binds to its cognate operator sequence on a double-stranded DNA oligonucleotide labeled at its 5'-end with tetramethylrhodamine (TAMRA). Phospho-FixJ binding to the DNA target slows down the rotational tumbling of the labeled DNA fragment resulting in an increased TAMRA fluorescence anisotropy. Blue-light illumination (blue bar) enhances the phosphatase activity of the LOV-HK resulting in rapid dephosphorylation of phospho-FixJ and unbinding from the labeled DNA fragment, hence rapidly decreasing the TAMRA fluorescence anisotropy signal. (B-E) Experimental results for SB1F1, SB2F1, SB2F1-I66R and YF1. All experiments were performed in triplicates (n = 3).

Table S1: SB2F1 crystal structures: data collection and refinement statistics.

Protein (State)	SB2F1 (Dark)	SB2F1 (Dark)	SB2F1 (illuminated)	SB2F1-I66R (Dark)	SB2F1-I66R (Light)
PDB ID	8A3U	8A6X	8A52	8A7F	8A7H
Beamline/Detector	ID29, ESRF (Grenoble, France)/DECTRIS PILATUS 6M-F (date 13-11-2015)	ID29, ESRF (Grenoble, France)/DECTRIS PILATUS 6M-F (date 13-11-2015)	ID30B, ESRF (Grenoble, France)/DECTRIS PILATUS3 6M (date 04-03-2016)	ID29, ESRF (Grenoble, France)/DECTRIS PILATUS 6M-F (date 21-06-2016)	ID29, ESRF (Grenoble, France)/DECTRIS PILATUS 6M-F (date 21-07-2016)
Wavelength (Å)/Monochromator	λ=0.97916/Silicon (1 1 1)	λ=0.97916/Silicon (1 1 1)	λ=0.97901/Silicon (1 1 1)	λ=0.97625 /Silicon (1 1 1)	λ=0.97625/Silicon (1 1 1)
Resolution range (Å), max.	45.65 - 2.327 (2.510 - 2.327)	45.43 - 2.452 (2.60 - 2.452)	45.252 - 2.461 (2.639 - 2.461)	45.345 - 2.71 (2.926 - 2.71)	45.64 - 3.145 (3.336 - 3.145)
Space group	$P\ 3_2\ 2\ 1$	$P\ 3_1\ 2\ 1$	P 3 2 1	$P\ 3_1\ 2\ 1$	$P\ 3_1\ 2\ 1$
Unit cell a=b, c (Å), α=β=90°, γ =120.0°	138.72 49.35	138.79 96.08	138.25 94.99	138.53 96.89	139.42 89.88
**Aimless/Staraniso**					
Total reflections	277759 (12332)	544226 (27231)	504669 (12873)	104836 (3851)	244700 (12736)
Unique reflections	13863 (693)	27082 (1369)	27319 (1387)	16593 (831)	12416 (621)
Multiplicity	20.0 (17.8)	20.1 (19.9)	18.5 (9.3)	6.3 (4.6)	19.7 (20.5)
Completeness (%) spherical	58.6 (14.6)	68.7 (21.9)	71.3 (19.4)	56.1 (13.7)	69.6 (21.6)
Completeness (%) ellipsoidal	92.0 (63.3)	95.5 (76.4)	94.3 (59.4)	92.1 (66.7)	94.0 (68.2)
Worst diffraction limit after cut-off (Å)	4,112	3,694	3.523	6,147	4,993
Mean I/sigma(I)	19.8 (1.5)	20.2 (1.6)	24.3 (1.4)	13.4 (1.4)	12.5 (1.5)
Wilson B-factor ($Å^2$)	75.13	77.7	75.18	86.45	109.89
CC (1/2)	0.999 (0.480)	0.999 (0.580)	0.999 (0.493)	1.0 (0.403)	0.996 (0.483)
R-merge	0.117 (2.260)	0.122(2.366)	0.102 (1.622)	0.069 (1.078)	0.163 (2.703)
R-meas	0.120 (2.327)	0.125 (2.429)	0.105 (1.718)	0.075 (1.216)	0.168 (2.771)
R-pim	0.027 (0.549)	0.028 (0.543)	0.024 (0.554)	0.029 (0.555)	0.038 (0.610)
Anomalous completeness (spherical)	58.6 (14.8)	68.7 (22.3)	71.3 (19.8)		
Anomalous completeness (ellipsoidal)	91.9 (63.1)	95.4 (6.5)	94.3 (59.4)		
Anomalous multiplicity	10.4 (9.1)	10.4 (10.1)	9.6 (4.7)		
CC(ano)	0.922 (0.029)	0.880 (0.026)	0.945 (-0.004)		
|DANO|/sd(DANO)	2.220 (0.819)	2.182 (0.787)	2.187 (0.796)		
**comment: phasing**	MSE-SAD phasing	MSE-SAD phasing	MSE-SAD phasing	MR with "8A6X"	MR with "8A6X"
**SAD-Phasing (SHELX-CDE)**					
fp/fdp	fp: -7.8705 fdp: 3.84174	fp: -7.8705 fdp: 3.84174	fp: -7.4025 fdp: 3.84046		
best shelxd solution	CC 52.83 CC(weak) 32.32 CFOM 85.15	CC 52.41 CC(weak) 25.22 CFOM 77.64	CC 55.81 CC(weak) 28.13 CFOM 83.94		
possible Se-sites (mol/asu)	9 (1)	18 (2)	18 (2)		
sites found (occ > 0.4)	4	9	15		
contrast shelxe	none	0.977	0.886		
rms coordinate differences to refined structure	not verified	0.71 for 16 pairs	0.66 for 16 pairs		
after phase improvement and extension FOM	0.481	0.574	0.589		
**Refinement (Phenix)**					
refined vs.	F^+_{obs}/F^-_{obs}	F^+_{obs}/F^-_{obs}	F^+_{obs}/F^-_{obs}	F_{obs}	F_{obs}
Resolution range (Å)	45.65 - 2.327 (2.423 - 2.327)	44.61 - 2.452 (2.54 - 2.452)	44.15 - 2.461 (2.549 - 2.461)	41.07 - 2.71 (2.807 - 2.71)	45.64 - 3.145 (3.257 - 3.145)
R-work	0.2320 (0.4171)	0.2259 (0.6222)	0.2432 (0.5106)	0.2283 (0.4655)	0.2533 (0.4156)
R-free	0.2797 (0.5910)	0.2667 (0.6726)	0.2952 (0.6977)	0.249	0.3056 (0.4467)
coordinate error (max.-likelihood based)	0.43	0.41	0.43	0.29	0.44
Number of non-hydrogen atoms	2891	5811	5784	5786	5756
macromolecules	2829	5687	5660	5662	5632
ligands	62	124	124	124	124
Protein residues	364	732	728	728	724
RMS (bonds)	0.004	0.008	0.005	0.005	0.005
RMS (angles)	0.64	Jan-17	0.77	0.84	0.93
Ramachandran favored (%)	97.79	97.66	97.38	98.9	97.08
Ramachandran outliers (%)	0	0	0	0	0
Clashscore	5.78	14.29	8.84	14.70	14.99
Average B-factor ($Å^2$)	120.42	99.96	90.21	127.48	104
macromolecules ($Å^2$)	120.6	100.25	90.51	127.78	104.28
ligands ($Å^2$)	112.33	86.6	76.51	113.47	91.57
Number of TLS groups	5	11	10	9	11
xtal conditions	10% PEG8K, 0.1M Citrate 6.0, 0.2M NaCl, 0.1M Ammonium Bromide; 1mM ATP, 2mM MgCl2	10% PEG8K, 0.1M Citrate 6.0, 0.2M NaCl, 0.1M Ammonium Trifluoroacetate; 1mM ATP,	6% PEG8K, 0.1M Na Citrate 6.15 0.2M NaCl; 1mM ATP, 2mM MgCl2	9% PEG8K, 0.1M Na-Citrate 5.7, 0.2M NaCl; 1mM ATP, 2mM MgCl	18% PEG 3,3K, 0.1M Bicine 9.3, 0.2M LiSO4; 1mM ATP, 2mM MgCl
cryo conditions	+ 15% PEG 8K, 20% Sucrose	+ 15% PEG 8K, 20% Sucrose	12% PEG8K, 0.1M Na Citrate 6.2, 0.2M NaCl, 30% PEG 200, 2mM MgCl2, 1.5mM ATP	+ 20%PEG200, ATP/Mg	+ 20%PEG200, ATP/Mg
Matthews coefficients $Å^3$/Da	3.2	3.12	3.2	3.13	2.94
solvent content %	61.63	60.63	61.63	66.8	58.25

* *Statistics for the highest-resolution shell are shown in parentheses*

Table S2: Root-mean-square deviation (RMSD) values between structural models.

**RMSD (Å) / *N*align**	SB2F1, dark PDB ID 8A6X	SB2F1-I66R, dark PDB ID 8A7F	SB2F1-I66R, light PDB ID 8A7H	YF1, dark PDB ID 4GCZ
SB2F1, dark PDB ID 8A3U	1.0918 / 364[a]			
SB2F1, Illuminated PDB ID 8A52	0.6424 / 728[b]			
SB2F1-I66R, dark PDB ID 8A7F	0.8972 / 728[b]<br>0.4561 / 236[c]			
SB2F1-I66R, light PDB ID 8A7H	1.6327 / 724[b]	1.9703 / 711[b]		
YF1, dark PDB ID 4GCZ	4.9704 / 302			
PpSB2-LOV, dark PDB ID 7A6P	3.2242 / 236[c]	3.2268 / 234[c]	3.086 / 235[c]	1.7395 / 242[c]
PpSB1-LOV, light PDB ID 3SW1	1.0522 / 247[c]	1.2189 / 251[c]	0.8582 / 239[c]	3.3312 / 242[c]
PpSB1-LOV, dark PDB ID 5J3W	2.9959 / 241[c]			1.5038 / 249[c]

RMSD: Root mean-square deviation, a measure of the average distance between equivalent Cα atoms in two superimposed protein structures.; *N*align: Number of aligned residues included in the RMSD calculation after superposition. Worse fits with higher RMSD values are highlighted in orange boxes. Calculations were performed using SSM superpose (*113*).

a: residues from one protein chain; b: residues from dimer; c: residues from the LOV core domain of the dimer.

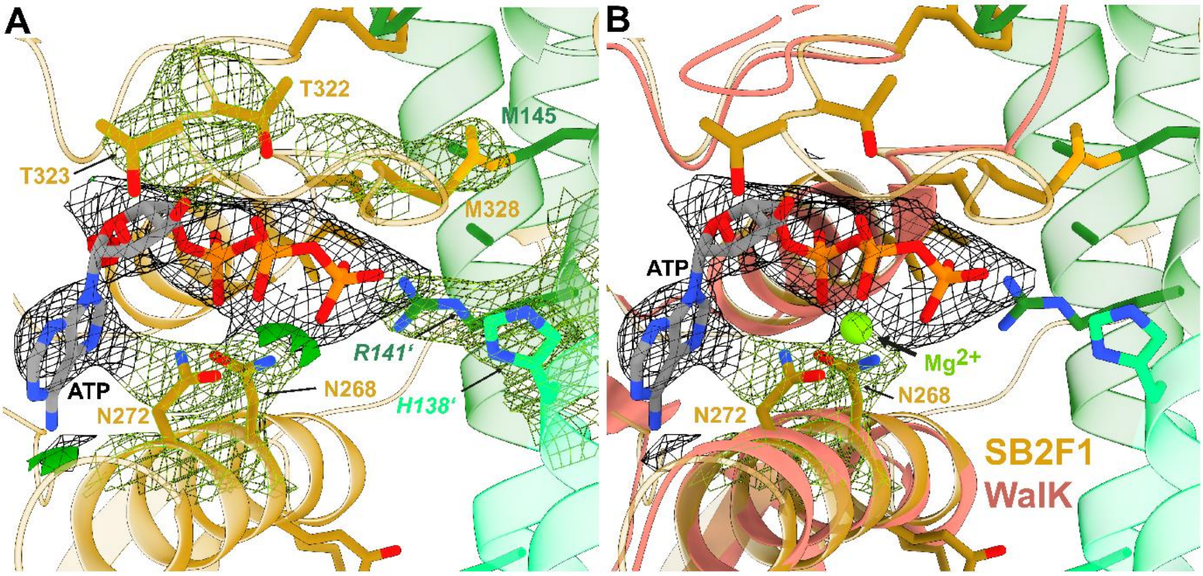

Figure S3: SB2F1 ATP-binding CA domain and CA-DHp interface. (A) SB2F1 dark-state structure and σ-A weighted 2mFo-DFc electron density map of the ATP molecule (black mesh) and the CA-DHp domain interface residues (light green mesh) contoured at 1.0 σ. The mFo-DFc map is shown as solid surfaces colored green (3.0 sigma) and red (-3.0 sigma) 2 Å from the ATP molecule. (B) Superposition of the SB2F1 CA-DHp domain interface (CA domain, in gold, DHp domain in dark and light green) and the WalK CA domain structure (PDB ID: 3SL2, salmon; chain A). The Mg^{2+} ion present in the WalK CA domain ATP binding site is shown as yellow green sphere. In addition, the 2mFo-DFc electron density map of the ATP molecule (black mesh) and electron density around the N272, N268 side chains (light green mesh) contoured at 1.0 σ is shown.

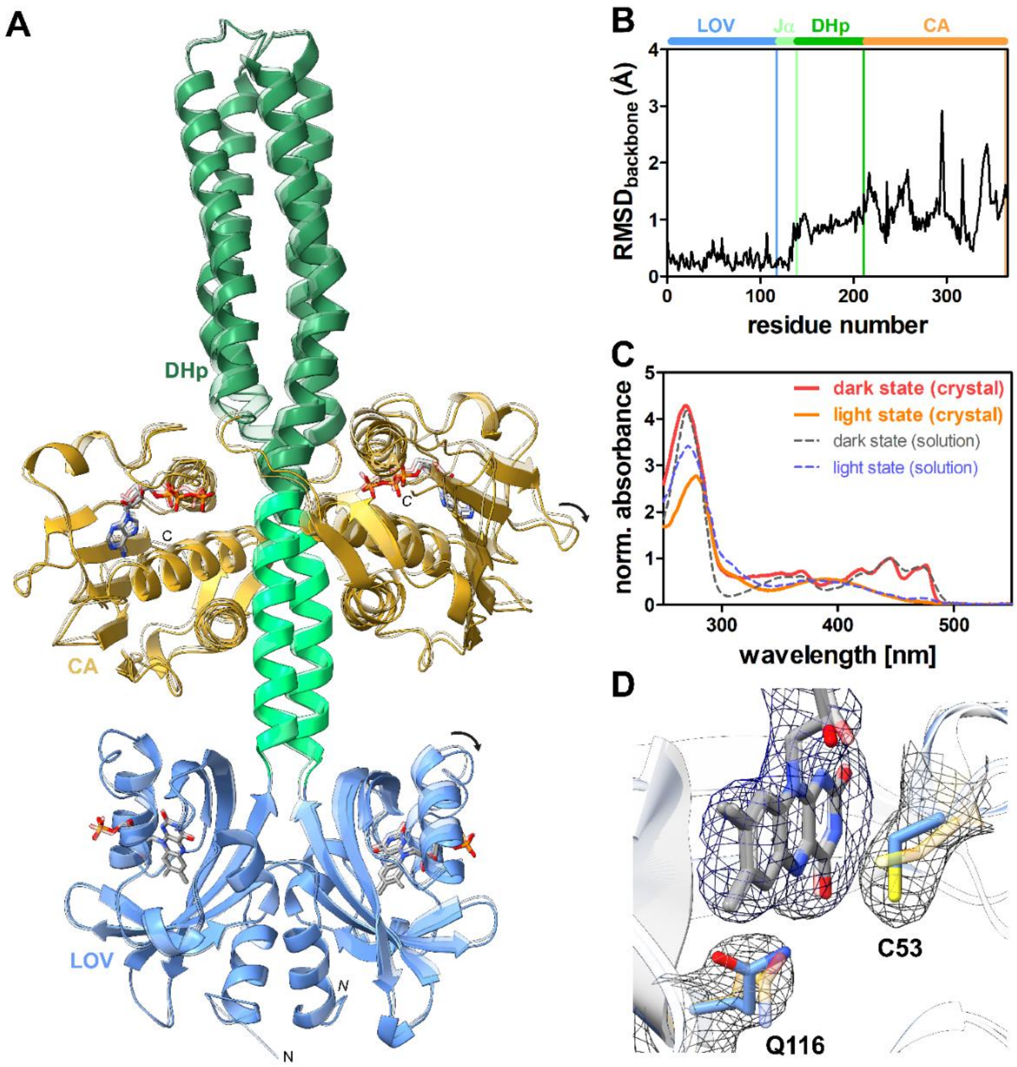

Figure S4: Comparison of SB2F1 dark- and SB2F1 illuminated-state structure. (A) Crystal structures of the SB2F1 illuminated state (solid ribbon) superimposed with the SB2-F1 dark state (transparent ribbon) with the domains colored as the follows: LOV – blue, DHp – light green and dark green (with the Jα-linker portion of the DHp α1-helix in light green and the remaining DHp helical spine in dark green) and CA – gold. FMN and ATP are shown in stick representation with carbon in grey, oxygen in red, nitrogen in blue and phosphorous in orange. Structures superimposed via chain A of the LOV-LOV dimer (RMSD over backbone atoms, residues 1-363; 0.942 Å). (B) Residues-wise RMSD of the backbone atoms. Bars above the graph highlight domain boundaries (see Figure 3). (C) Single-crystal microspectrometry data. Absorbance spectra recorded for the dark-grown SB2F1 crystal (red solid line), the illuminated dark grown crystal (solid orange line) and solution dark- and light-state spectra (dashed grey and blue lines). All spectra were normalized to the 450 nm (dark state) and 390 nm (light state) absorbance band, respectively. Light-state (D) σ-A weighted 2mFo-DFc electron density map of the FMN chromophore, cysteine 53 and glutamine 116 of the SB2F1 illuminated-state structure contoured at 1.0 σ. The corresponding side chains and the FMN molecule are shown in stick representation with carbon atoms in blue, while the corresponding dark-state conformation is shown with carbon atoms in orange, respectively.

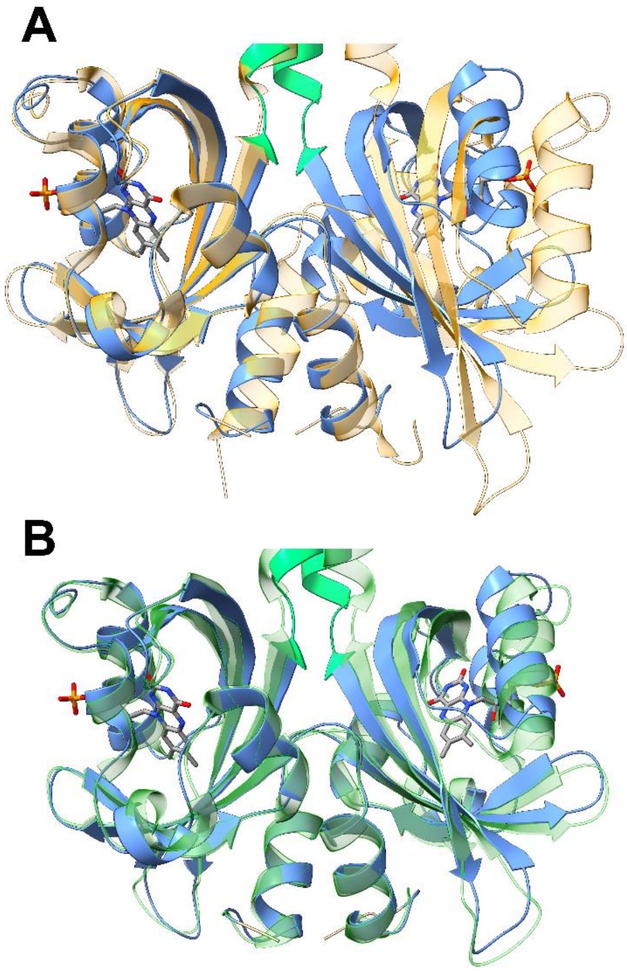

Figure S5: SB2F1-I66R dark-state LOV-LOV dimer arrangement. Superposition of the SB2F1-I66R dark state LOV-LOV dimer (blue solid ribbon) and (A) the PpSB2-LOV dark state structure (PDB-ID: 7A6P, (*53*))(transparent orange ribbon) (backbone RMSD over residues 1-118; 3.23 Å) or (B) the PpSB1-LOV light-state structure (PDB-ID: 3SW1, (*45*))(transparent dark green ribbon) (backbone RMSD over residues 1-118; 1.21 Å). The FMN chromophore is shown in stick representation with carbon atoms in grey, oxygen in red, nitrogen in blue and phosphorous in orange.

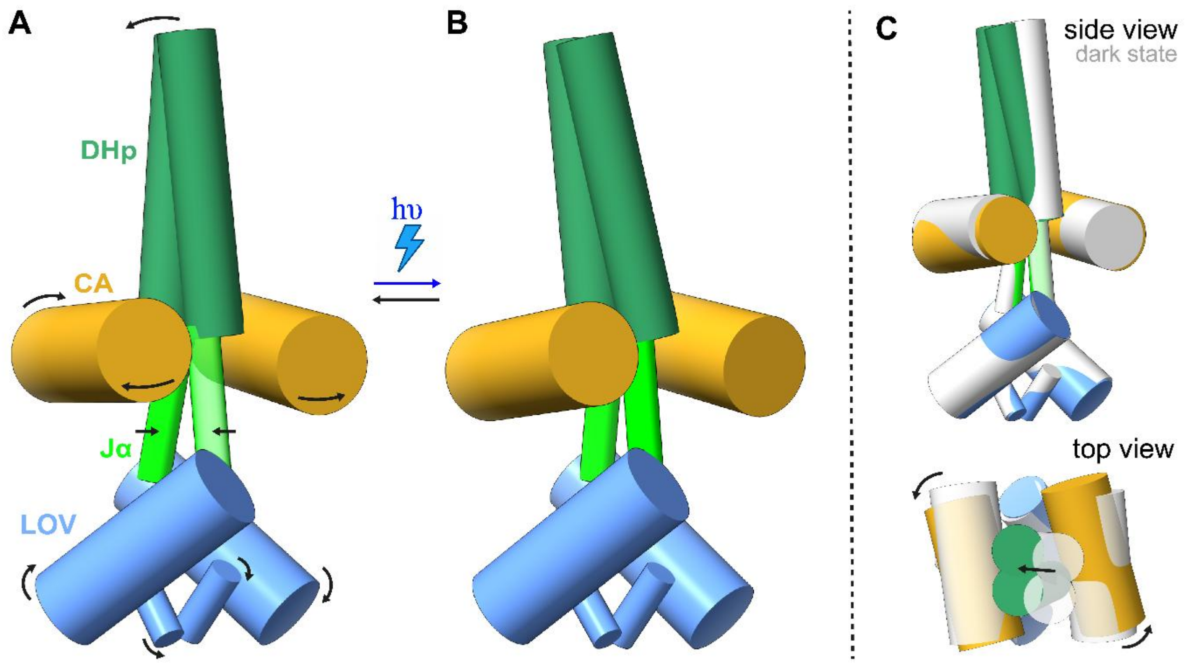

Figure S6: Overview of global structural differences between SB2F1-I66R in the dark state (A) and light state (B). The structures were modelled by fitting defined axis into the A'α-helix, the LOV core, the Jα-helix, the DHp domain and the CA domain of both chains using ChimeraX 1.9. (C) Side view and top view if superposed dark-state (grey) and light-state structures (colored as follows: LOV – blue, DHp – light green and dark green (with the Jα-linker portion of the DHp α1-helix in light green and the remaining DHp helical spine in dark green) and CA – gold). Arrows in (A) mark movements of sub-structures (A'α and Jα-helix) and subdomains (LOV, DHp, CA) relative to the dark structure. Structural changes in the N-terminal sensory module include a minor rotation of the chain B in LOV dimer relative to chain A. This results in a decreased helix-crossing angle for the Jα helix portion of the DHp domain (12.8°) as compared to the dark state (15.6°). Consequently, the CA domains undergo a translational/rotational movement, resulting in a less parallel arrangement in the light-state dimer (crossing angle CA-CA; 14.6°) vs the dark state (8.8°). The most pronounced structural change appears to be the DHp domain displacement, which adopts a more bent-conformation in the light state.

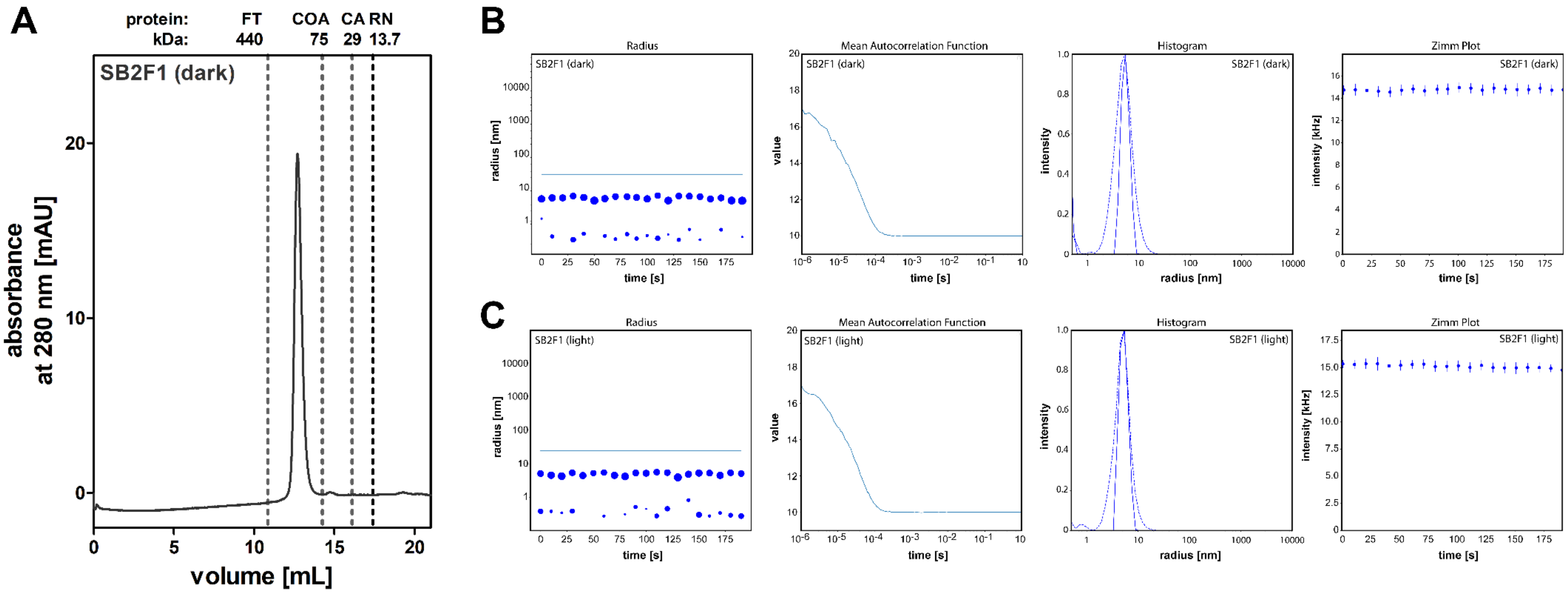

Supporting Figure S7: Analytical size exclusion (SEC) and DLS analyses of SB2F1. (A) SEC chromatogram of the dark adapted SB2F1 protein. Superdex 200 Increase 10/300 GL columns, Buffer: 20 mM Tris at pH 8.0, 40 mM NaCl. Dashed lines mark the elution peak maxima of different reference proteins of known molecular weight, that were used for calibration. FT: ferritin, 440 kDa, COA: conalbumin, 75 kDa; CA: carbonic anhydrase, 29 kDa; RN: Ribonulease A, 13.7 kDa. (B, C) Dynamic light scattering analysis of SB2F1 in the dark (B) and light state (C). Analysis yielded an R_h = 4.91 ± 0.54 nm (PDI 31.9 %) (dark state) and R_h = 4.96 ± 0.49 nm (PDI 27.1 %). All samples (conc. 1 mg mL^{-1}) in 20 mm Tris pH 8.0, 40 mM NaCl contained 1.5 mM ATP and 3 mM $MgCl_2$. Analyses were performed at 20°C in the dark or after 30 sec illumination with blue light. As described in the Methods, DLS data analysis was performed using the SpectroCrystal software provided by the manufacturer.

Table S3: Summary of SB2F1 SAXS studies.

	**SB2F1 dark state** ATP + $MgCl_2$)	**SB2F1 light state** (ATP + $MgCl_2$)
Calculated MW for monomer/dimer [kDa]	42.8 / 85.6	
SEC MW [kDa]	117	n.d.[1]
SAXS MW from I_0 [kDa]	64.3	66.7
SAXS MW from Porod volume [kDa]	80.0	78.8
DLS R_h [nm]	4.91	4.96
Crystal structure R_h [nm][2]	4.00	n.a.[3]
Crystal structure R_g [nm][2]	3.4	n.a.[3]
Guinier R_g from SAXS [nm][4]	3.96 ± 0.01	3.73 ± 0.01
D_{max} from SAXS [nm]	13.7	12.8
χ^2 for the ATP-bound SB2F1 dark[5]	23.332	**4.655**
χ^2 for the ATP-bound SB2F1 illuminated[5]	24.347	5.428
χ^2 for the ATP-bound SB2F1-I66R dark[5]	23.485	4.703
χ^2 for the ATP-bound SB2F1-I66R light[5]	24.424	6.315
χ^2 for the YF1[5,6]	**5.637**	8.902

1: n.d. – not determined; 2: calculated using HydroPro10 (*114*) 3: n.a. – not available; 4: limits $0.23 < q \times Rg < 1.25$ (dark state dataset), limits: $0.26 < q \times Rg < 1.29$ (light state dataset); 5: χ^2 value for the best fitting structure are highlighted in bold, respectively; 6: PDB ID: 4GCZ (*33*)

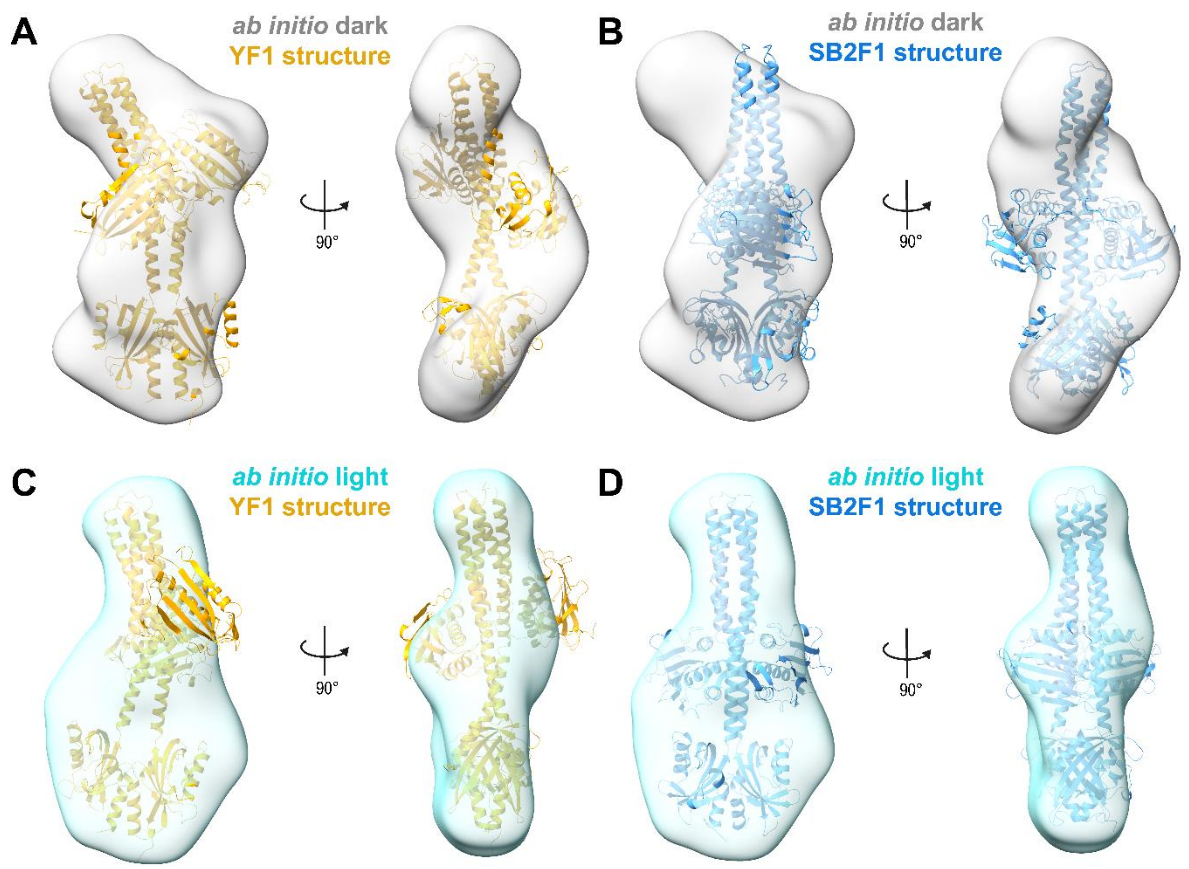

Figure S8: *Ab initio* models evaluation. Averaged and filtered *ab initio* models (transparent surface; grey: dark state, cyan: light state) superimposed with the YF1 dark state structure (PDB-ID: 4GCZ, (*33*); orange cartoon) and SB2F1 dark state structure (blue cartoon). Maps contoured at 1σ. For clarity FMN and ADP/ATP ligands are not shown. (A) Dark-state *ab initio* model vs. YF1 dark state structure (PDB-ID 4GCZ). (B) Dark-state *ab initio* model vs. SB2F1 dark state structure (PDB-ID 8A6X). (C) Light-state *ab initio* model vs. YF1 dark state structure (PDB-ID 4GCZ). (B) Light-state *ab initio* model vs. SB2F1 dark state structure (PDB-ID 8A6X)

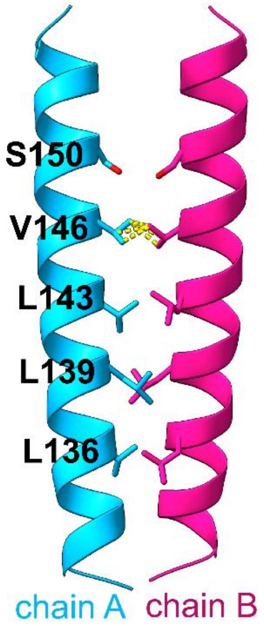

Figure S9: Structural model of a canonical YF1 Jα coiled coil. The two chains of the coiled-coil dimer are shown as cyan and pink cartoon. Residues of the hydrophobic interface are shown in stick representation. Clashes between V146 of opposing chains in the canonical coiled-coil model, as inferred from clash analysis by ChimeraX (*108*), are highlighted (dashed yellow lines).

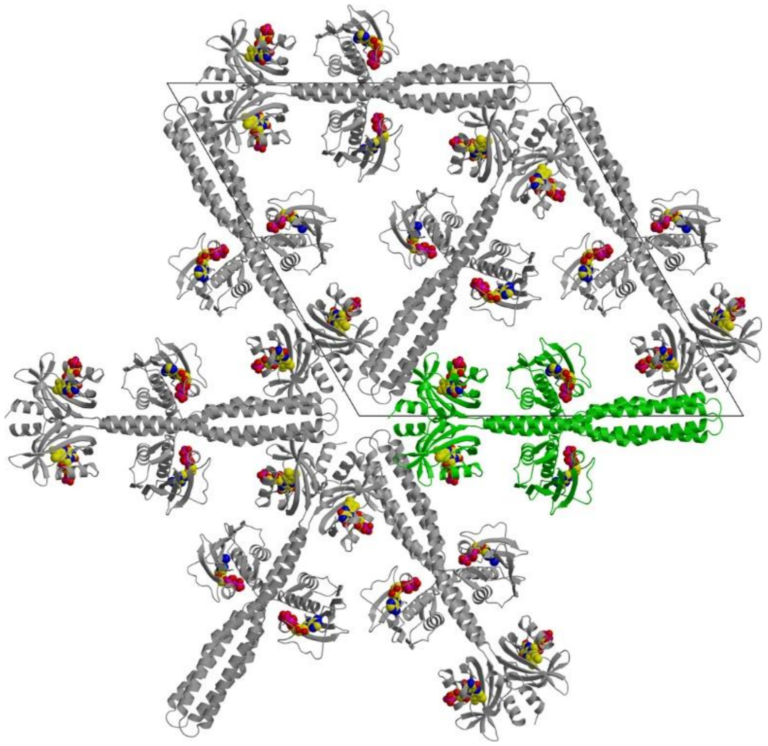

Figure S10: Crystal packing in SB2F1 crystals. Several symmetry-related SB2F1 dimers are depicted as ribbon representations in gray, with one dimer highlighted in green for clarity. Notably, the CA domains are positioned within solvent channels, allowing greater flexibility due to the absence of direct crystal contacts. Ligands ATP and FMN are shown as space-filling spheres, with carbon in yellow, oxygen in red, nitrogen in blue, and phosphorus in magenta.

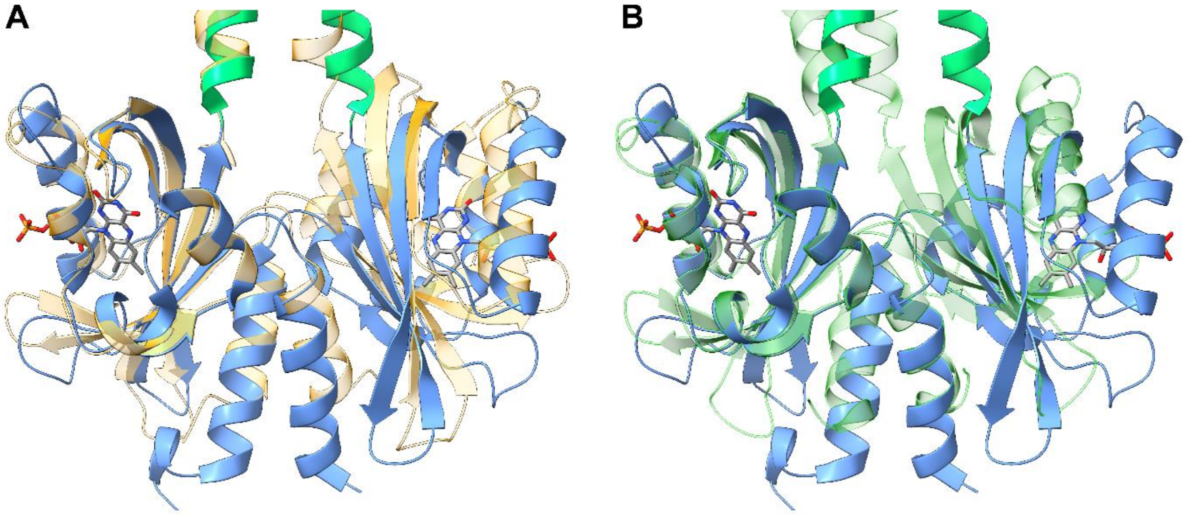

Figure S11: YF1 dark-state LOV-LOV dimer arrangement. Superposition of the YF1 dark-state LOV-LOV dimer (blue solid ribbon)(PDB-ID: 4GCZ; (*33*) and (A) the PpSB1-LOV dark-state structure (PDB-ID: 5J3W, (*47*))(transparent orange ribbon) (3.37 Å backbone RMSD over residues 13-127 of YF1) or (B) the PpSB1-LOV light-state structure (PDB-ID: 3SW1, (*45*))(transparent dark green ribbon) (6.96 Å backbone RMSD backbone RMSD over residues 13-127 of YF1). The FMN chromophores are shown in stick representation with carbon atoms in grey, oxygen in red, nitrogen in blue and phosphorous in orange.

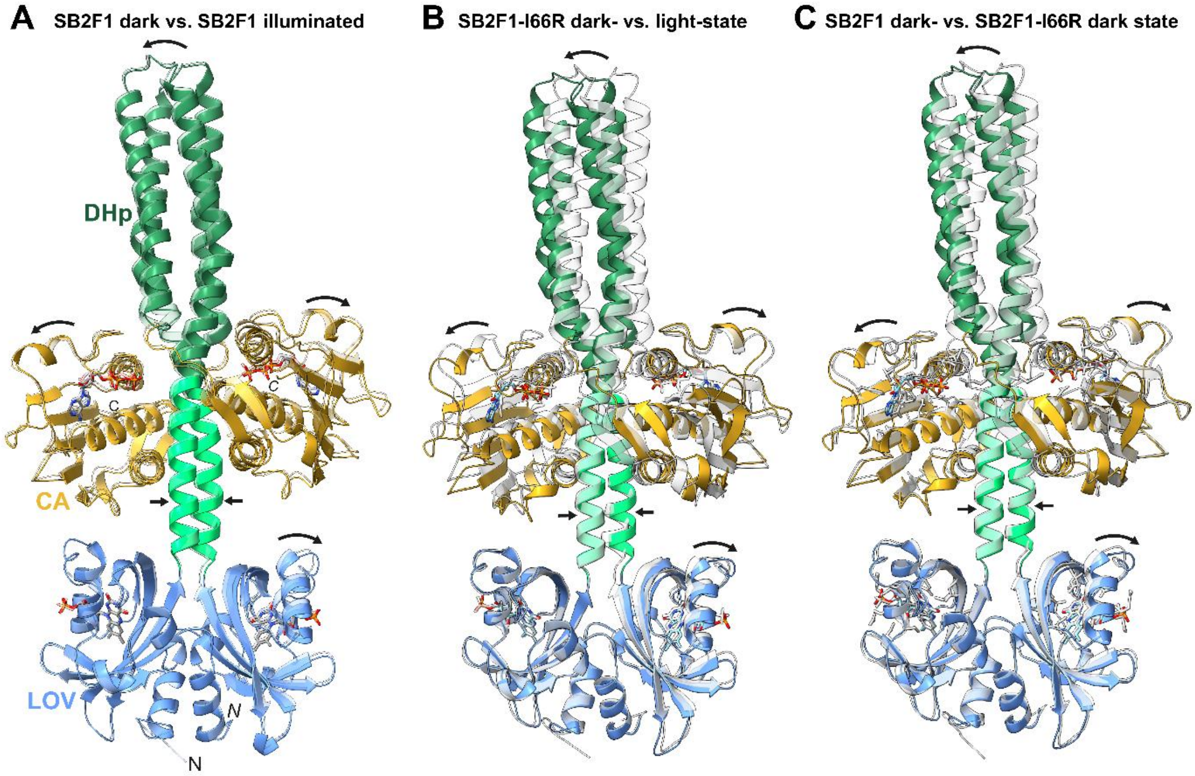

Figure S12: Comparison of the light-dependent structural changes of SB2F1 and SB2F1-I66R. Superposition of (A) SB2F1 dark-state (grey transparent ribbon) and SB2F1 illuminated-state structure (solid ribbon, domain coloring), (B) SB2F1-I66R dark state (grey transparent ribbon) and SB2F1-I66R light-state structure (solid ribbon, domain coloring) and (C) SB2F1 dark-state (grey transparent ribbon) and SB2F1-I66R dark-state structure (solid ribbon, domain coloring). Domain coloring according to architecture as follows: LOV – blue, DHp – light green and dark green (with the Jα-linker portion of the DHp α1-helix in light green and the remaining DHp helical spine in dark green) and CA – gold. Arrows mark domain motions. The FMN chromophore and the ATP ligand are shown in stick representation with carbon atoms in grey, oxygen in red, nitrogen in blue and phosphorous in orange.

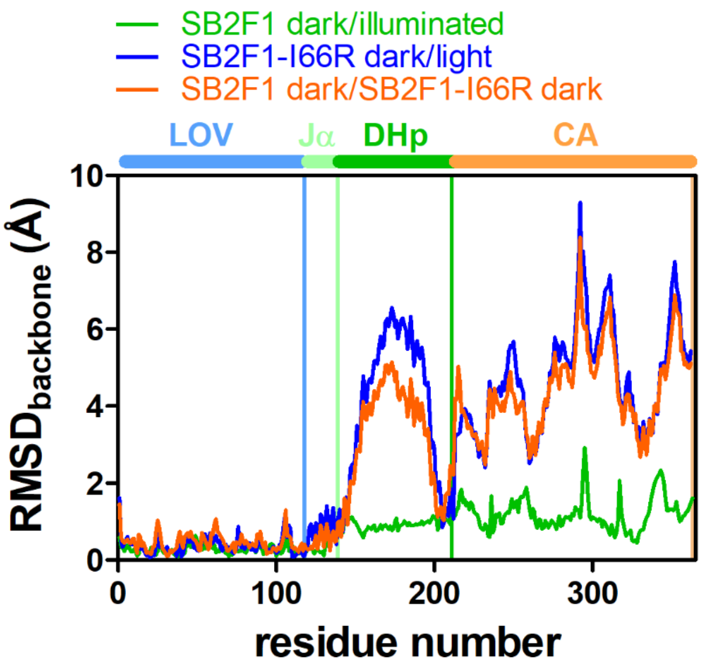

Figure S13: RMSD-based comparison of the SB2F1 dark and SB2F1-I66R dark-/light-state structures. Residues-wise RMSD of the backbone atoms for the comparison shown in Supporting Figure S12 A-C. Bars above the graph highlight domain boundaries (see Figure 3).

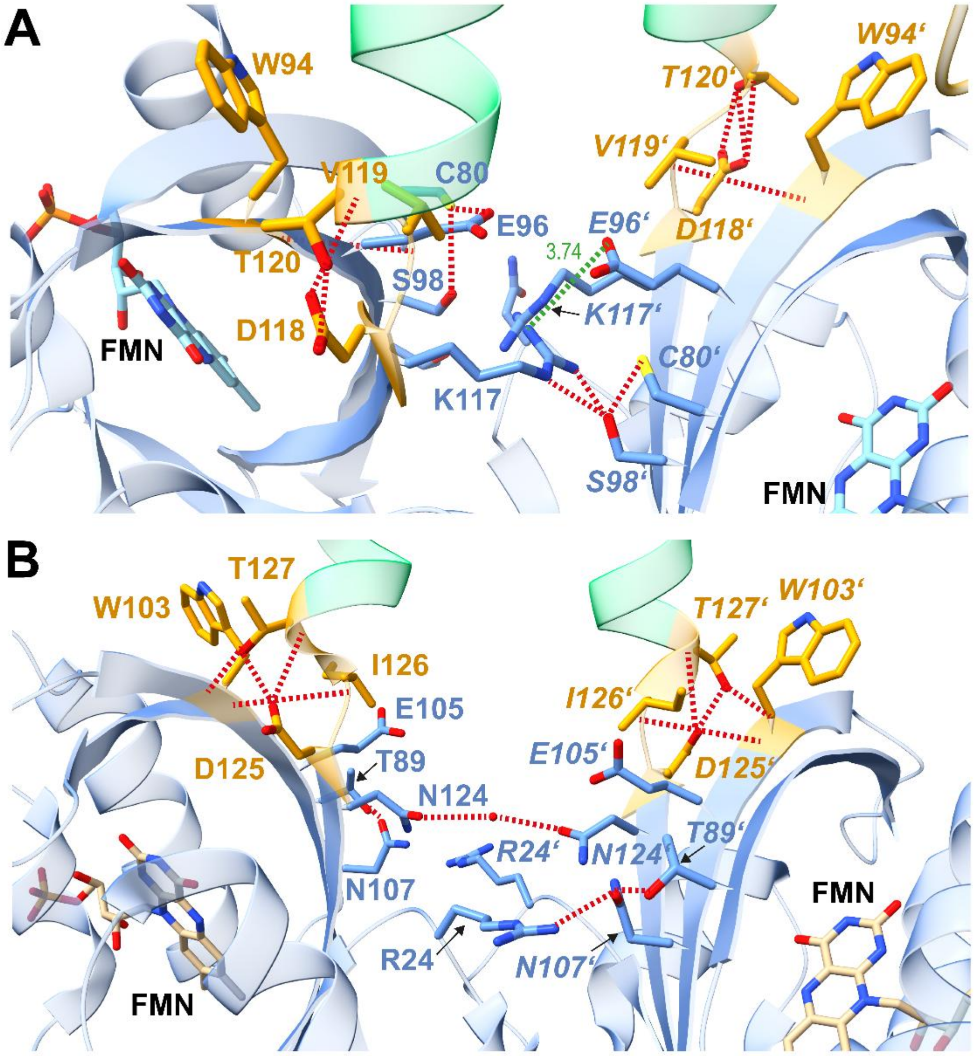

Figure S14: Interface analyses of SB2F1 and YF1. LOV-LOV dimer interface of SB2F1 (A) and YF1 (B), shown as ribbon diagrams with LOV domains (blue), Jα helices (light green), and DVT (SB2F1) or DIT (YF1) motifs (orange). Key inter- and intra-subunit residues are shown as sticks with matching carbon colors. H-bonds (<3.2 Å) and salt bridges (<4.0 Å) are indicated as dashed red and green lines, respectively.

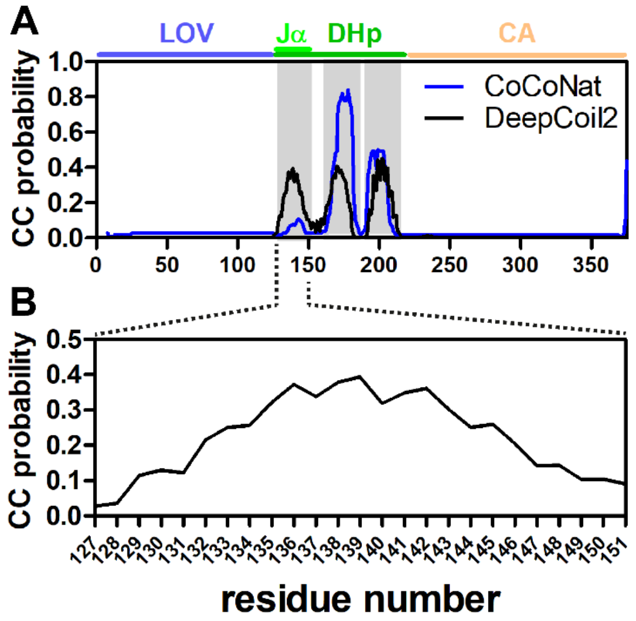

Figure S15: Results of coiled-coil probability prediction for the YF1 sequence. (A) The probability for the presence of canonical coiled-coil heptad repeat patterns was predicted based on sequence using the neural-network based coiled-coil prediction tools DeepCoil2 (data shown in black) (*76*) and CoCoNat (data shown in blue) (*77*). Lines above the graph mark domain boundaries of the LOV, DHp (containing the Jα element) and CA domains. (B) Coiled-coil propensity, predicted by DeepCoil2 (*76*) for the Jα-helix segment of YF1.

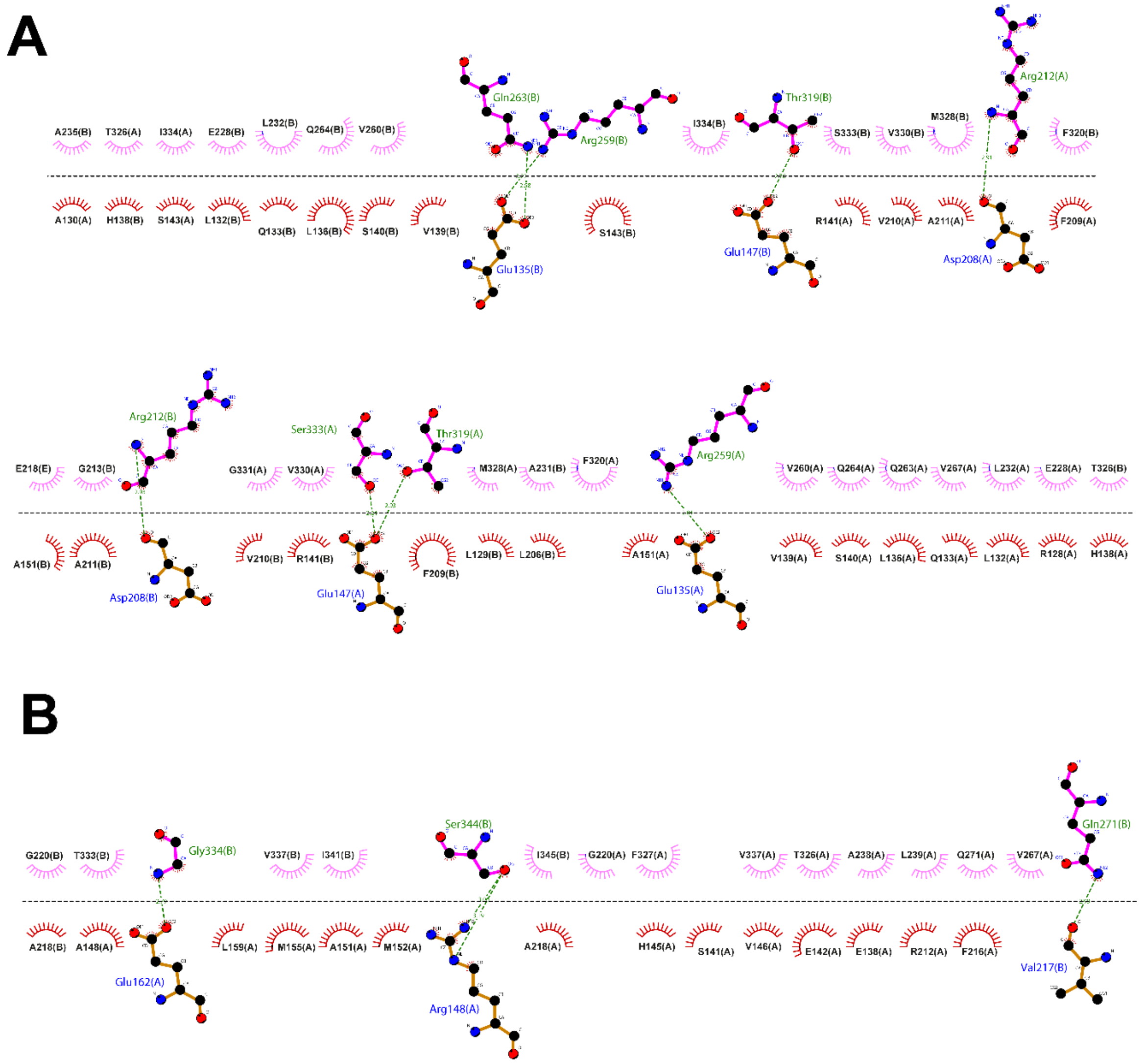

Figure S16: DimPlot analysis of the SB2F1 and YF1 Jα-DHp ··· CA domain interactions. DimPlot analysis for (A) SB2F1 dark-state structure; DHp and CA domain defined as follows. DHp: 120-211 (phosphor-accepting His) CA: 212-363. And (B) YF1 (PDB: 4GCZ, (*33*)); DHp and CA domain defined as follows. DHp: 127-218 (phosphor-accepting His) CA: 220-370. All analyses were performed using the LigPlot+ v2.2. software tool (*109*).

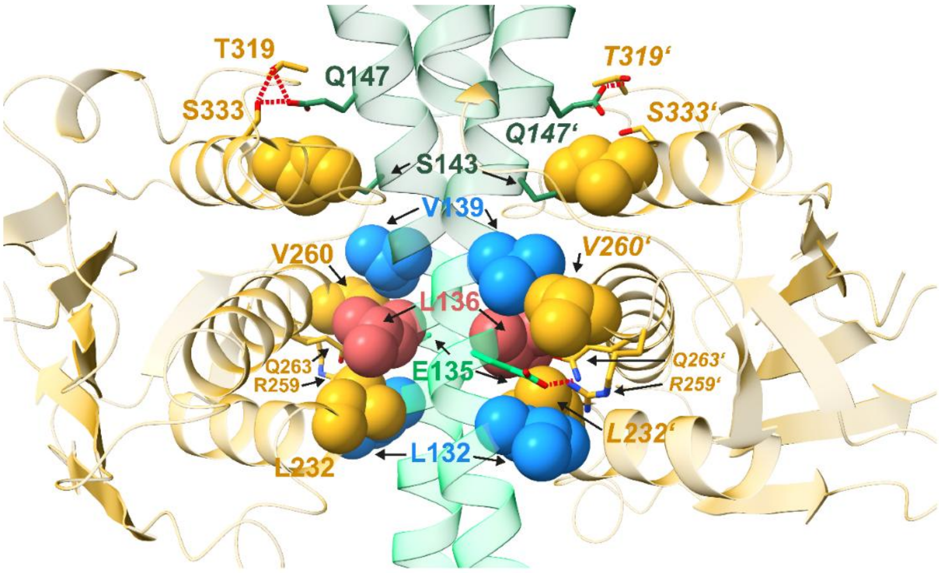

Figure S17: SB2F1 DHp–CA interface. Hydrophobic residues are shown as spheres and H-bonding/polar residues as sticks (H-bonds <3.2 Å; red dashed lines). In all panels atom coloring as follows: C–domain coloring, O–red, N–blue, P–orange. Key contacts include hydrophobic interactions between the exposed hydrophobic Jα core residues L132, L136, V139 and hydrophobic residues on the CA domain (mostly L232, V260) as well as polar contacts between residues within the Jα-helix (E135) / DHp spine (Q147) and the CA domain (Q263, R259, T319, S333)

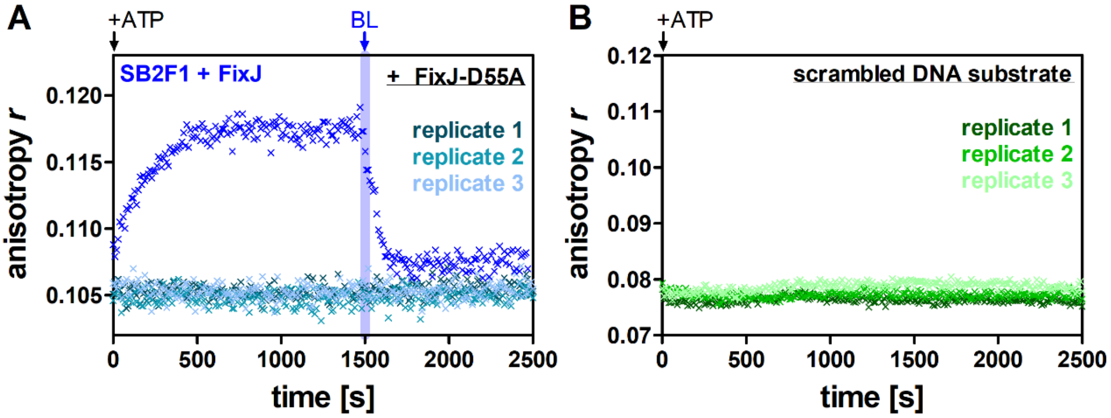

Figure S18: Evaluation of fluorescence anisotropy-based assay for functional characterization of SHKs. For a description of the assay principle see Figure S2. (A) To verify that the increase in fluorescence anisotropy arises from specific binding of phosphorylated FixJ to the TAMRA-labeled double-stranded DNA molecule containing the phospho-FixJ operator site, we performed the histidine kinase assay with SB2F1 and a variant of FixJ, in which the phosphor-accepting D55 was mutated to alanine (FixJ-D55A). Dark equilibrated SB2F1 samples were incubated in the dark with FixJ-D55A and the TAMRA-labeled DNA substrate, containing the FixK2 operator sequence. The measurements were conducted in triplicate (n =3; data shown in shades of cyan). As a positive control the same experiment was performed with the SB2F1/FixJ pair (data shown in dark blue) To start the kinase reaction, ATP was added and TAMRA fluorescence anisotropy was monitored over time. An increase in anisotropy reflects kinase activity, i.e., resulting in the formation of phospho-FixJ and its binding to the DNA. Subsequent illumination with blue-light (indicated by a blue bar) caused a decrease of the anisotropy, consistent with net phosphatase activity. (B) Histidine kinase assay performed with a scrambled TAMRA-labeled DNA substrate, having the same overall length and GC content, but random sequence. Measurements was performed using dark adapted SB2F1 samples incubated in the dark with FixJ and the scrambled DNA substrate. The reactions were started by the addition of ATP. All measurements were conducted in triplicate (n =3; data shown in shades of green). Please note that, while no increase in fluorescence anisotropy over time is seen, indicating that phospho-FixJ does not bind to the scrambled DNA substrate, the basal anisotropy of the TAMRA-labeled scrambled DNA substrate is lower as compared to the basal anisotropy of the TAMRA-labeled DNA substrate containing the FixK2 operator sequence (compare Figure 2, panel F of the main manuscript).

REFERENCES

1. E. J. Capra, M. T. Laub, Evolution of two-component signal transduction systems. *Annu. Rev. Microbiol.* **66**, 325–347 (2012).

2. R. Gao, A. M. Stock, Biological insights from structures of two-component proteins. *Annu. Rev. Microbiol.* **63**, 133–154 (2009).

3. D. Beier, R. Gross, Regulation of bacterial virulence by two-component systems. *Curr. Opin. Microbiol.* **9**, 143–152 (2006).

4. C. Fabret, J. A. Hoch, A two-component signal transduction system essential for growth of *Bacillus subtilis*: Implications for anti-infective therapy. *J. Bacteriol.* **180**, 6375–6383 (1998).

5. M. D. Senadheera, B. Guggenheim, G. A. Spatafora, Y. C. Huang, J. Choi, D. C. Hung, J. S. Treglown, S. D. Goodman, R. P. Ellen, D. G. Cvitkovitch, A VicRK signal transduction system in *Streptococcus mutans* affects gtfBCD, gbpB, and ftf expression, biofilm formation, and genetic competence development. *J. Bacteriol.* **187**, 4064–4076 (2005).

6. A. M. Stock, V. L. Robinson, P. N. Goudreau, Two-component signal transduction. *Annu. Rev. Biochem.* **69**, 183–215 (2000).

7. A. R. Tierney, P. N. Rather, Roles of two-component regulatory systems in antibiotic resistance. *Future Microbiol.* **14**, 533–552 (2019).

8. R. Cai, W. He, J. Zhang, R. Liu, Z. Yin, X. Zhang, C. Sun, Blue light promotes zero-valent sulfur production in a deep-sea bacterium. *EMBO J.* **42**, e112514 (2023).

9. V. P. Conforte, J. Rinaldi, H. R. Bonomi, A. Festa, D. Garacoche, S. Foscaldi, E. Castagnaro, A. A. Vojnov, F. Malamud, *Xanthomonas campestris* pv. campestris regulates virulence mechanisms by sensing blue light. *Photochem. Photobiol. Sci.* **24**, 327–342 (2025).

10. R. Foreman, A. Fiebig, S. Crosson, The LovK-LovR two-component system is a regulator of the general stress pathway in *Caulobacter crescentus*. *J. Bacteriol.* **194**, 3038–3049 (2012).

11. T. E. Swartz, T. S. Tseng, M. A. Frederickson, G. Paris, D. J. Comerci, G. Rajashekara, J. G. Kim, M. B. Mudgett, G. A. Splitter, R. A. Ugalde, F. A. Goldbaum, W. R. Briggs, R. A. Bogomolni, Blue-light-activated histidine kinases: Two-component sensors in bacteria. *Science* **317**, 1090–1093 (2007).

12. H. Aiba, T. Mizuno, Phosphorylation of a bacterial activator protein, OmpR, by a protein kinase, EnvZ, stimulates the transcription of the ompF and ompC genes in *Escherichia coli*. *FEBS Lett.* **261**, 19–22 (1990).

13. M. Liu, G. Hao, Z. Li, Y. Zhou, R. Garcia-Sillas, J. Li, H. Wang, B. Kan, J. Zhu, CitAB two-component system-regulated citrate utilization contributes to *Vibrio cholerae* competitiveness with the gut microbiota. *Infect. Immun.* **87**, e00746-18 (2019).

14. T. Yoshida, S. Phadtare, M. Inouye, Functional and structural characterization of EnvZ, an osmosensing histidine kinase of *E. coli*. *Methods Enzymol.* **423**, 184–202 (2007).

15. J. Cheung, W. A. Hendrickson, Structural analysis of ligand stimulation of the histidine kinase NarX. *Structure* **17**, 190–201 (2009).

16. E. B. Goh, P. J. Bledsoe, L. L. Chen, P. Gyaneshwar, V. Stewart, M. M. Igo, Hierarchical control of anaerobic gene expression in *Escherichia coli* K-12: The nitrate-responsive NarX-NarL regulatory system represses synthesis of the fumarate-responsive DcuS-DcuR regulatory system. *J. Bacteriol.* **187**, 4890–4899 (2005).

17. G. L. Hazelbauer, J. J. Falke, J. S. Parkinson, Bacterial chemoreceptors: High-performance signaling in networked arrays. *Trends Biochem. Sci.* **33**, 9–19 (2008).

18. M. A. Gilles-Gonzalez, G. S. Ditta, D. R. Helinski, A haemoprotein with kinase activity encoded by the oxygen sensor of *Rhizobium meliloti*. *Nature* **350**, 170–172 (1991).

19. N. B. Ivleva, T. Gao, A. C. LiWang, S. S. Golden, Quinone sensing by the circadian input kinase of the cyanobacterial circadian clock. *Proc. Natl. Acad. Sci. U.S.A.* **103**, 17468–17473 (2006).

20. G. S. A. Wright, A. Saeki, T. Hikima, Y. Nishizono, T. Hisano, M. Kamaya, K. Nukina, H. Nishitani, H. Nakamura, M. Yamamoto, S. V. Antonyuk, S. S. Hasnain, Y. Shiro, H. Sawai, Architecture of the complete oxygen-sensing FixL-FixJ two-component signal transduction system. *Sci. Signal.* **11**, eaaq0825 (2018).

21. W. Wu, P. Kumar, C. A. Brautigam, S. C. Tso, H. R. Baniasadi, D. L. Kober, M. A. Gilles-Gonzalez, Structures of the multi-domain oxygen sensor DosP: Remote control of a c-di-GMP phosphodiesterase by a regulatory PAS domain. *Nat. Commun.* **15**, 9653 (2024).

22. F. D. Russo, T. J. Silhavy, The essential tension: Opposed reactions in bacterial two-component regulatory systems. *Trends Microbiol.* **1**, 306–310 (1993).

23. A. Möglich, R. A. Ayers, K. Moffat, Design and signaling mechanism of light-regulated histidine kinases. *J. Mol. Biol.* **385**, 1433–1444 (2009).

24. A. Möglich, Signal transduction in photoreceptor histidine kinases. *Protein Sci.* **28**, 1923–1946 (2019).

25. S. H. Bhoo, S. J. Davis, J. Walker, B. Karniol, R. D. Vierstra, Bacteriophytochromes are photochromic histidine kinases using a biliverdin chromophore. *Nature* **414**, 776–779 (2001).

26. T. N. Malla, C. Hernandez, S. Muniyappan, D. Menendez, D. Bizhga, J. H. Mendez, P. Schwander, E. A. Stojkovic, M. Schmidt, Photoreception and signaling in bacterial phytochrome revealed by single-particle cryo-EM. *Sci. Adv.* **10**, eadq0653 (2024).

27. E. Multamaki, R. Nanekar, D. Morozov, T. Lievonen, D. Golonka, W. Y. Wahlgren, B. Stucki-Buchli, J. Rossi, V. P. Hytonen, S. Westenhoff, J. A. Ihalainen, A. Möglich, H. Takala, Comparative analysis of two paradigm bacteriophytochromes reveals opposite functionalities in two-component signaling. *Nat. Commun.* **12**, 4394 (2021).

28. L. H. Otero, S. Foscaldi, G. T. Antelo, G. L. Rosano, S. Sirigu, S. Klinke, L. A. Defelipe, M. Sanchez-Lamas, G. Battocchio, V. Conforte, A. A. Vojnov, L. M. G. Chavas, F. A. Goldbaum, M. A. Mroginski, J. Rinaldi, H. R. Bonomi, Structural basis for the Pr-Pfr long-range signaling

mechanism of a full-length bacterial phytochrome at the atomic level. *Sci. Adv.* **7**, eabh1097 (2021).

29. L. Sauthof, M. Szczepek, A. Schmidt, A. Bhowmick, M. Dasgupta, M. J. Mackintosh, S. Gul, F. D. Fuller, R. Chatterjee, I. D. Young, N. Michael, N. A. Heyder, B. Bauer, A. Koch, I. Bogacz, I. S. Kim, P. S. Simon, A. Butryn, P. Aller, V. U. Chukhutsina, J. M. Baxter, C. D. M. Hutchison, D. Liebschner, B. Poon, N. K. Sauter, M. D. Miller, N. P. George, Jr., R. Alonso-Mori, M. S. Hunter, A. Batyuk, S. Owada, K. Tono, R. Tanaka, J. J. van Thor, N. Krauss, T. Lamparter, A. S. Brewster, I. Schapiro, A. M. Orville, V. K. Yachandra, J. Yano, P. Hildebrandt, J. F. Kern, P. Scheerer, Serial-femtosecond crystallography reveals how a phytochrome variant couples chromophore and protein structural changes. *Sci. Adv.* **11**, eadp2665 (2025).

30. M. K. Shankar, L. Grunewald, W. Y. Wahlgren, B. Stucki-Buchli, A. Nimmrich, M. Kurttila, A. L. Fischer, G. Salvadori, A. Cellini, P. Maj, A. L. Anindya, E. Claesson, F. Luo, T. N. Malla, S. Pandey, T. Tosha, N. Nipawan, S. Owada, K. Tono, R. Tanaka, E. A. Stojkovic, D. Mozorov, P. Myllyperkio, T. Kumpulainen, H. Takala, M. Schmidt, J. A. Ihalainen, S. Westenhoff, Ultrafast, remote-controlled protonation reaction enables structural changes in a phytochrome. *Sci. Adv.* **11**, eady0499 (2025).

31. H. R. Bonomi, D. M. Posadas, G. Paris, C. Carrica Mdel, M. Frederickson, L. I. Pietrasanta, R. A. Bogomolni, A. Zorreguieta, F. A. Goldbaum, Light regulates attachment, exopolysaccharide production, and nodulation in *Rhizobium leguminosarum* through a LOV-histidine kinase photoreceptor. *Proc. Natl. Acad. Sci. U.S.A.* **109**, 12135–12140 (2012).

32. E. B. Purcell, D. Siegal-Gaskins, D. C. Rawling, A. Fiebig, S. Crosson, A photosensory two-component system regulates bacterial cell attachment. *Proc. Natl. Acad. Sci. U.S.A.* **104**, 18241–18246 (2007).

33. R. P. Diensthuber, M. Bommer, T. Gleichmann, A. Möglich, Full-length structure of a sensor histidine kinase pinpoints coaxial coiled coils as signal transducers and modulators. *Structure* **21**, 1127–1136 (2013).

34. J. Rinaldi, I. Fernandez, H. Shin, G. Sycz, S. Gunawardana, I. Kumarapperuma, J. M. Paz, L. H. Otero, M. L. Cerutti, A. Zorreguieta, Z. Ren, S. Klinke, X. Yang, F. A. Goldbaum, Dimer asymmetry and light activation mechanism in brucella blue-light sensor histidine kinase. *MBio* **12**, e00264-21 (2021).

35. G. Rivera-Cancel, W. H. Ko, D. R. Tomchick, F. Correa, K. H. Gardner, Full-length structure of a monomeric histidine kinase reveals basis for sensory regulation. *Proc. Natl. Acad. Sci. U.S.A.* **111**, 17839–17844 (2014).

36. A. Bury, K. J. Hellingwerf, Design, characterization and in vivo functioning of a light-dependent histidine protein kinase in the yeast Saccharomyces cerevisiae. *AMB Express* **8**, 53 (2018).

37. A. Levskaya, A. A. Chevalier, J. J. Tabor, Z. B. Simpson, L. A. Lavery, M. Levy, E. A. Davidson, A. Scouras, A. D. Ellington, E. M. Marcotte, C. A. Voigt, Synthetic biology: Engineering *Escherichia coli* to see light. *Nature* **438**, 441–442 (2005).

38. S. S. M. Meier, E. Multamaki, A. T. Ranzani, H. Takala, A. Möglich, Leveraging the histidine kinase-phosphatase duality to sculpt two-component signaling. *Nat. Commun.* **15**, 4876 (2024).

39. R. Ohlendorf, R. R. Vidavski, A. Eldar, K. Moffat, A. Möglich, From dusk till dawn: One-plasmid systems for light-regulated gene expression. *J. Mol. Biol.* **416**, 534–542 (2012).

40. J. J. Tabor, A. Levskaya, C. A. Voigt, Multichromatic control of gene expression in *Escherichia coli*. *J. Mol. Biol.* **405**, 315–324 (2011).

41. A. Losi, E. Polverini, B. Quest, W. Gärtner, First evidence for phototropin-related blue-light receptors in prokaryotes. *Biophys. J.* **82**, 2627–2634 (2002).

42. W. Gong, B. Hao, S. S. Mansy, G. Gonzalez, M. A. Gilles-Gonzalez, M. K. Chan, Structure of a biological oxygen sensor: A new mechanism for heme-driven signal transduction. *Proc. Natl. Acad. Sci. U.S.A.* **95**, 15177–15182 (1998).

43. K. S. Conrad, C. C. Manahan, B. R. Crane, Photochemistry of flavoprotein light sensors. *Nat. Chem. Biol.* **10**, 801–809 (2014).

44. J. Herrou, S. Crosson, Function, structure and mechanism of bacterial photosensory LOV proteins. *Nat. Rev. Microbiol.* **9**, 713–723 (2011).

45. F. Circolone, J. Granzin, K. Jentzsch, T. Drepper, K. E. Jaeger, D. Willbold, U. Krauss, R. Batra-Safferling, Structural basis for the slow dark recovery of a full-length LOV protein from *Pseudomonas putida*. *J. Mol. Biol.* **417**, 362–374 (2012).

46. S. M. Harper, L. C. Neil, K. H. Gardner, Structural basis of a phototropin light switch. *Science* **301**, 1541–1544 (2003).

47. K. Röllen, J. Granzin, V. Panwalkar, V. Arinkin, R. Rani, R. Hartmann, U. Krauss, K. E. Jaeger, D. Willbold, R. Batra-Safferling, Signaling states of a short blue-light photoreceptor protein PpSB1-LOV revealed from crystal structures and solution NMR spectroscopy. *J. Mol. Biol.* **428**, 3721–3736 (2016).

48. J. P. Zayner, C. Antoniou, T. R. Sosnick, The amino-terminal helix modulates light-activated conformational changes in AsLOV2. *J. Mol. Biol.* **419**, 61–74 (2012).

49. J. M. Christie, P. Reymond, G. K. Powell, P. Bernasconi, A. A. Raibekas, E. Liscum, W. R. Briggs, Arabidopsis NPH1: A flavoprotein with the properties of a photoreceptor for phototropism. *Science* **282**, 1698–1701 (1998).

50. K. Röllen, J. Granzin, R. Batra-Safferling, A. M. Stadler, Small-angle X-ray scattering study of the kinetics of light-dark transition in a LOV protein. *PLOS ONE* **13**, e0200746 (2018).

51. A. T. Vaidya, C. H. Chen, J. C. Dunlap, J. J. Loros, B. R. Crane, Structure of a light-activated LOV protein dimer that regulates transcription. *Sci. Signal.* **4**, ra50 (2011).

52. K. Jentzsch, A. Wirtz, F. Circolone, T. Drepper, A. Losi, W. Gärtner, K. E. Jaeger, U. Krauss, Mutual exchange of kinetic properties by extended mutagenesis in two short LOV domain proteins from *Pseudomonas putida*. *Biochemistry* **48**, 10321–10333 (2009).

53. V. Arinkin, J. Granzin, U. Krauss, K. E. Jaeger, D. Willbold, R. Batra-Safferling, Structural determinants underlying the adduct lifetime in the LOV proteins of *Pseudomonas putida*. *FEBS J.* **288**, 4955–4972 (2021).

54. J. E. Hart, S. Sullivan, P. Hermanowicz, J. Petersen, L. A. Diaz-Ramos, D. J. Hoey, J. Labuz, J. M. Christie, Engineering the phototropin photocycle improves photoreceptor performance and plant biomass production. *Proc. Natl. Acad. Sci. U.S.A.* **116**, 12550–12557 (2019).

55. J. Dietler, R. Gelfert, J. Kaiser, V. Borin, C. Renzl, S. Pilsl, A. T. Ranzani, A. Garcia de Fuentes, T. Gleichmann, R. P. Diensthuber, M. Weyand, G. Mayer, I. Schapiro, A. Möglich, Signal transduction in light-oxygen-voltage receptors lacking the active-site glutamine. *Nat. Commun.* **13**, 2618 (2022).

56. M. Bocola, U. Schwaneberg, K. E. Jaeger, U. Krauss, Light-induced structural changes in a short light, oxygen, voltage (LOV) protein revealed by molecular dynamics simulations-implications for the understanding of LOV photoactivation. *Front. Mol. Biosci.* **2**, 55 (2015).

57. Y. Zhao, Y. Zhang, M. Sun, Q. Zheng, A theoretical study on the signal transduction process of bacterial photoreceptor PpSB1 based on the Markov state model. *Phys. Chem. Chem. Phys.* **23**, 2398–2405 (2021).

58. S. Crosson, K. Moffat, Structure of a flavin-binding plant photoreceptor domain: Insights into light-mediated signal transduction. *Proc. Natl. Acad. Sci. U.S.A.* **98**, 2995–3000 (2001).

59. A. S. Halavaty, K. Moffat, N- and C-terminal flanking regions modulate light-induced signal transduction in the LOV2 domain of the blue light sensor phototropin 1 from *Avena sativa*. *Biochemistry* **46**, 14001–14009 (2007).

60. A. Möglich, K. Moffat, Structural basis for light-dependent signaling in the dimeric LOV domain of the photosensor YtvA. *J. Mol. Biol.* **373**, 112–126 (2007).

61. J. S. Parkinson, E. C. Kofoid, Communication modules in bacterial signaling proteins. *Annu. Rev. Genet.* **26**, 71–112 (1992).

62. R. Celikel, V. H. Veldore, I. Mathews, K. M. Devine, K. I. Varughese, ATP forms a stable complex with the essential histidine kinase WalK (YycG) domain. *Acta Crystallogr. D Biol. Crystallogr.* **68**, 839–845 (2012).

63. S. Endres, J. Granzin, F. Circolone, A. Stadler, U. Krauss, T. Drepper, V. Svensson, E. Knieps-Grünhagen, A. Wirtz, A. Cousin, P. Tielen, D. Willbold, K. E. Jaeger, R. Batra-Safferling, Structure and function of a short LOV protein from the marine phototrophic bacterium *Dinoroseobacter shibae*. *BMC Microbiol.* **15**, 30 (2015).

64. R. Fedorov, I. Schlichting, E. Hartmann, T. Domratcheva, M. Fuhrmann, P. Hegemann, Crystal structures and molecular mechanism of a light-induced signaling switch: The Phot-LOV1 domain from *Chlamydomonas reinhardtii*. *Biophys. J.* **84**, 2474–2482 (2003).

65. B. D. Zoltowski, C. Schwerdtfeger, J. Widom, J. J. Loros, A. M. Bilwes, J. C. Dunlap, B. R. Crane, Conformational switching in the fungal light sensor Vivid. *Science* **316**, 1054–1057 (2007).

66. A. Ganguly, W. Thiel, B. R. Crane, Glutamine amide flip elicits long distance allosteric responses in the LOV protein vivid. *J. Am. Chem. Soc.* **139**, 2972–2980 (2017).

67. A. I. Nash, W. H. Ko, S. M. Harper, K. H. Gardner, A conserved glutamine plays a central role in LOV domain signal transmission and its duration. *Biochemistry* **47**, 13842–13849 (2008).

68. V. Arinkin, J. Granzin, K. E. Jaeger, D. Willbold, U. Krauss, R. Batra-Safferling, Conserved signal transduction mechanisms and dark recovery kinetic tuning in the pseudomonadaceae short light, oxygen, voltage (LOV) protein family. *J. Mol. Biol.* **436**, 168458 (2024).

69. K. Henzler-Wildman, D. Kern, Dynamic personalities of proteins. *Nature* **450**, 964–972 (2007).

70. J. Monod, J. Wyman, J. P. Changeux, On the nature of allosteric transitions: A plausible model. *J. Mol. Biol.* **12**, 88–118 (1965).

71. X. Yao, M. K. Rosen, K. H. Gardner, Estimation of the available free energy in a LOV2-Jα photoswitch. *Nat. Chem. Biol.* **4**, 491–497 (2008).

72. D. Strickland, X. Yao, G. Gawlak, M. K. Rosen, K. H. Gardner, T. R. Sosnick, Rationally improving LOV domain-based photoswitches. *Nat. Methods* **7**, 623–626 (2010).

73. O. Berntsson, R. P. Diensthuber, M. R. Panman, A. Bjorling, E. Gustavsson, M. Hoernke, A. J. Hughes, L. Henry, S. Niebling, H. Takala, J. A. Ihalainen, G. Newby, S. Kerruth, J. Heberle, M. Liebi, A. Menzel, R. Henning, I. Kosheleva, A. Moglich, S. Westenhoff, Sequential conformational transitions and α-helical supercoiling regulate a sensor histidine kinase. *Nat. Commun.* **8**, 284 (2017).

74. C. Engelhard, R. P. Diensthuber, A. Möglich, R. Bittl, Blue-light reception through quaternary transitions. *Sci. Rep.* **7**, 1385 (2017).

75. A. Möglich, R. A. Ayers, K. Moffat, Structure and signaling mechanism of Per-ARNT-Sim domains. *Structure* **17**, 1282–1294 (2009).

76. J. Ludwiczak, A. Winski, K. Szczepaniak, V. Alva, S. Dunin-Horkawicz, DeepCoil-a fast and accurate prediction of coiled-coil domains in protein sequences. *Bioinformatics* **35**, 2790–2795 (2019).

77. G. Madeo, C. Savojardo, M. Manfredi, P. L. Martelli, R. Casadio, CoCoNat: A novel method based on deep learning for coiled-coil prediction. *Bioinformatics* **39**, btad495 (2023).

78. P. Kumar, D. N. Woolfson, Socket2: A program for locating, visualizing and analyzing coiled-coil interfaces in protein structures. *Bioinformatics* **37**, 4575–4577 (2021).

79. E. Krissinel, K. Henrick, Inference of macromolecular assemblies from crystalline state. *J. Mol. Biol.* **372**, 774–797 (2007).

80. R. Ohlendorf, C. H. Schumacher, F. Richter, A. Möglich, Library-aided probing of linker determinants in hybrid photoreceptors. *ACS Synth. Biol.* **5**, 1117–1126 (2016).

81. T. Gleichmann, R. P. Diensthuber, A. Möglich, Charting the signal trajectory in a light-oxygen-voltage photoreceptor by random mutagenesis and covariance analysis. *J. Biol. Chem.* **288**, 29345–29355 (2013).

82. A. M. Stadler, E. Knieps-Grünhagen, M. Bocola, W. Lohstroh, M. Zamponi, U. Krauss, Photoactivation reduces side-chain dynamics of a LOV photoreceptor. *Biophys. J.* **110**, 1064–1074 (2016).

83. A. M. Stadler, J. Schneidewind, M. Zamponi, E. Knieps-Grünhagen, S. Gholami, U. Schwaneberg, I. Rivalta, M. Garavelli, M. D. Davari, K. E. Jaeger, U. Krauss, Ternary complex formation and photoactivation of a photoenzyme results in altered protein dynamics. *J. Phys. Chem. B* **123**, 7372–7384 (2019).

84. F. Jacob-Dubuisson, A. Mechaly, J. M. Betton, R. Antoine, Structural insights into the signalling mechanisms of two-component systems. *Nat. Rev. Microbiol.* **16**, 585–593 (2018).

85. E. Lesne, E. M. Krammer, E. Dupre, C. Locht, M. F. Lensink, R. Antoine, F. Jacob-Dubuisson, Balance between coiled-coil stability and dynamics regulates activity of BvgS sensor kinase in *Bordetella*. *MBio* **7**, e02089 (2016).

86. W. Y. Wahlgren, E. Claesson, I. Tuure, S. Trillo-Muyo, S. Bodizs, J. A. Ihalainen, H. Takala, S. Westenhoff, Structural mechanism of signal transduction in a phytochrome histidine kinase. *Nat. Commun.* **13**, 7673 (2022).

87. R. M. Horton, S. N. Ho, J. K. Pullen, H. D. Hunt, Z. Cai, L. R. Pease, Gene splicing by overlap extension. *Methods Enzymol.* **217**, 270–279 (1993).

88. R. M. Horton, H. D. Hunt, S. N. Ho, J. K. Pullen, L. R. Pease, Engineering hybrid genes without the use of restriction enzymes: Gene splicing by overlap extension. *Gene* **77**, 61–68 (1989).

89. F. W. Studier, Protein production by auto-induction in high density shaking cultures. *Protein Expr. Purif.* **41**, 207–234 (2005).

90. T. Mathes, C. Vogl, J. Stolz, P. Hegemann, In vivo generation of flavoproteins with modified cofactors. *J. Mol. Biol.* **385**, 1511–1518 (2009).

91. S. Doublie, Production of selenomethionyl proteins in prokaryotic and eukaryotic expression systems. *Methods Mol. Biol.* **363**, 91–108 (2007).

92. R. Rani, K. Jentzsch, J. Lecher, R. Hartmann, D. Willbold, K. E. Jaeger, U. Krauss, Conservation of dark recovery kinetic parameters and structural features in the pseudomonadaceae “short” light, oxygen, voltage (LOV) protein family: Implications for the design of LOV-based optogenetic tools. *Biochemistry* **52**, 4460–4473 (2013).

93. E. F. Yee, R. P. Diensthuber, A. T. Vaidya, P. P. Borbat, C. Engelhard, J. H. Freed, R. Bittl, A. Moglich, B. R. Crane, Signal transduction in light-oxygen-voltage receptors lacking the adduct-forming cysteine residue. *Nat. Commun.* **6**, 10079 (2015).

94. J. Hennemann, R. S. Iwasaki, T. N. Grund, R. P. Diensthuber, F. Richter, A. Möglich, Optogenetic control by pulsed illumination. *Chembiochem* **19**, 1296–1304 (2018).

95. D. von Stetten, T. Giraud, P. Carpentier, F. Sever, M. Terrien, F. Dobias, D. H. Juers, D. Flot, C. Mueller-Dieckmann, G. A. Leonard, D. de Sanctis, A. Royant, In crystallo optical spectroscopy (icOS) as a complementary tool on the macromolecular crystallography beamlines of the ESRF. *Acta Crystallogr. D Biol. Crystallogr.* **71**, 15–26 (2015).

96. D. de Sanctis, A. Beteva, H. Caserotto, F. Dobias, J. Gabadinho, T. Giraud, A. Gobbo, M. Guijarro, M. Lentini, B. Lavault, T. Mairs, S. McSweeney, S. Petitdemange, V. Rey-Bakaikoa, J. Surr, P. Theveneau, G. A. Leonard, C. Mueller-Dieckmann, ID29: A high-intensity highly automated ESRF beamline for macromolecular crystallography experiments exploiting anomalous scattering. *J. Synchrotron Radiat.* **19**, 455–461 (2012).

97. A. A. McCarthy, R. Barrett, A. Beteva, H. Caserotto, F. Dobias, F. Felisaz, T. Giraud, M. Guijarro, R. Janocha, A. Khadrouche, M. Lentini, G. A. Leonard, M. Lopez Marrero, S. Malbet-Monaco, S. McSweeney, D. Nurizzo, G. Papp, C. Rossi, J. Sinoir, C. Sorez, J. Surr, O. Svensson, U. Zander, F. Cipriani, P. Theveneau, C. Mueller-Dieckmann, ID30B – A versatile beamline for macromolecular crystallography experiments at the ESRF. *J. Synchrotron Radiat.* **25**, 1249–1260 (2018).

98. G. P. Bourenkov, A. N. Popov, Optimization of data collection taking radiation damage into account. *Acta Crystallogr. D Biol. Crystallogr.* **66**, 409–419 (2010).

99. W. Kabsch, Xds. *Acta Crystallogr. D. Biol. Crystallogr.* **66**, 125–132 (2010).

100. M. D. Winn, C. C. Ballard, K. D. Cowtan, E. J. Dodson, P. Emsley, P. R. Evans, R. M. Keegan, E. B. Krissinel, A. G. Leslie, A. McCoy, S. J. McNicholas, G. N. Murshudov, N. S. Pannu, E. A. Potterton, H. R. Powell, R. J. Read, A. Vagin, K. S. Wilson, Overview of the CCP4 suite and current developments. *Acta Crystallogr. D Biol. Crystallogr.* **67**, 235–242 (2011).

101. P. D. Adams, P. V. Afonine, G. Bunkoczi, V. B. Chen, I. W. Davis, N. Echols, J. J. Headd, L. W. Hung, G. J. Kapral, R. W. Grosse-Kunstleve, A. J. McCoy, N. W. Moriarty, R. Oeffner, R. J. Read, D. C. Richardson, J. S. Richardson, T. C. Terwilliger, P. H. Zwart, PHENIX: A comprehensive Python-based system for macromolecular structure solution. *Acta Crystallogr. D Biol. Crystallogr.* **66**, 213–221 (2010).

102. P. Emsley, K. Cowtan, Coot: Model-building tools for molecular graphics. *Acta Crystallogr. D Biol. Crystallogr.* **60**, 2126–2132 (2004).

103. P. Pernot, A. Round, R. Barrett, A. De Maria Antolinos, A. Gobbo, E. Gordon, J. Huet, J. Kieffer, M. Lentini, M. Mattenet, C. Morawe, C. Mueller-Dieckmann, S. Ohlsson, W. Schmid, J. Surr, P. Theveneau, L. Zerrad, S. McSweeney, Upgraded ESRF BM29 beamline for SAXS on macromolecules in solution. *J. Synchrotron Radiat.* **20**, 660–664 (2013).

104. M. D. Tully, J. Kieffer, M. E. Brennich, R. Cohen Aberdam, J. B. Florial, S. Hutin, M. Oscarsson, A. Beteva, A. Popov, D. Moussaoui, P. Theveneau, G. Papp, J. Gigmes, F. Cipriani, A. McCarthy, C. Zubieta, C. Mueller-Dieckmann, G. Leonard, P. Pernot, BioSAXS at European synchrotron radiation facility – Extremely brilliant source: BM29 with an upgraded source, detector, robot, sample environment, data collection and analysis software. *J. Synchrotron Radiat.* **30**, 258–266 (2023).

105. K. Manalastas-Cantos, P. V. Konarev, N. R. Hajizadeh, A. G. Kikhney, M. V. Petoukhov, D. S. Molodenskiy, A. Panjkovich, H. D. T. Mertens, A. Gruzinov, C. Borges, C. M. Jeffries, D. I. Svergun, D. Franke, ATSAS 3.0: Expanded functionality and new tools for small-angle scattering data analysis. *J. Appl. Cryst.* **54**, 343–355 (2021).

106. W. Wriggers, Conventions and workflows for using Situs. *Acta Crystallogr. D Biol. Crystallogr.* **68**, 344–351 (2012).

107. V. S. The PyMOL Molecular Graphics System, LLC.

108. E. C. Meng, T. D. Goddard, E. F. Pettersen, G. S. Couch, Z. J. Pearson, J. H. Morris, T. E. Ferrin, UCSF chimeraX: Tools for structure building and analysis. *Protein Sci.* **32**, e4792 (2023).

109. R. A. Laskowski, M. B. Swindells, LigPlot+: Multiple ligand-protein interaction diagrams for drug discovery. *J. Chem. Inf. Model.* **51**, 2778–2786 (2011).

110. W. Kabsch, A solution for the best rotation to relate two sets of vectors. *Acta Crystallogr.* **32**, 922–923 (1976).

111. C. W. Wood, D. N. Woolfson, CCBuilder 2.0: Powerful and accessible coiled-coil modeling. *Protein Sci.* **27**, 103–111 (2018).

112. W. Kabsch, C. Sander, Dictionary of protein secondary structure: Pattern recognition of hydrogen-bonded and geometrical features. *Biopolymers* **22**, 2577–2637 (1983).

113. E. Krissinel, K. Henrick, Secondary-structure matching (SSM), a new tool for fast protein structure alignment in three dimensions. *Acta Crystallogr. D Biol. Crystallogr.* **60**, 2256–2268 (2004).

114. A. Ortega, D. Amoros, J. Garcia de la Torre, Prediction of hydrodynamic and other solution properties of rigid proteins from atomic- and residue-level models. *Biophys. J.* **101**, 892–898 (2011).
